# High current density electroreduction of CO_2_ into formate with tin oxide nanospheres

**DOI:** 10.1038/s41598-022-11890-6

**Published:** 2022-05-19

**Authors:** Thuy-Duong Nguyen-Phan, Leiming Hu, Bret H. Howard, Wenqian Xu, Eli Stavitski, Denis Leshchev, August Rothenberger, Kenneth C. Neyerlin, Douglas R. Kauffman

**Affiliations:** 1 National Energy Technology Laboratory, 626 Cochrans Mill Road, P.O. Box 10940, Pittsburgh, PA 15236-0940 USA; 2 NETL Support Contractor, 626 Cochrans Mill Road, P.O. Box 10940, Pittsburgh, PA 15236-0940 USA; 3 National Renewable Energy Laboratory, Golden, CO 80401 USA; 4 X-Ray Science Division, Advanced Photon Source, Argonne National Laboratory, Argonne, IL 60439 USA; 5 Photon Sciences Division, National Synchrotron Light Source II, Brookhaven National Laboratory, Upton, NY 11973 USA

**Keywords:** Electrocatalysis, Catalysis, Electrocatalysis, Electrochemistry, Electrocatalysis, Chemistry, Electrochemistry, Electrocatalysis, Materials science, Materials for energy and catalysis, Nanoscale materials

## Abstract

In this study, we demonstrate three-dimensional (3D) hollow nanosphere electrocatalysts for CO_2_ conversion into formate with excellent H-Cell performance and industrially-relevant current density in a 25 cm^2^ membrane electrode assembly electrolyzer device. Varying calcination temperature maximized formate production via optimizing the crystallinity and particle size of the constituent SnO_2_ nanoparticles. The best performing SnO_2_ nanosphere catalysts contained ~ 7.5 nm nanocrystals and produced 71–81% formate Faradaic efficiency (FE) between −0.9 V and −1.3 V vs. the reversible hydrogen electrode (RHE) at a maximum formate partial current density of 73 ± 2 mA cm_geo_^−2^ at −1.3 V vs. RHE. The higher performance of nanosphere catalysts over SnO_2_ nanoparticles and commercially-available catalyst could be ascribed to their initial structure providing higher electrochemical surface area and preventing extensive nanocrystal growth during CO_2_ reduction. Our results are among the highest performance reported for SnO_2_ electrocatalysts in aqueous H-cells. We observed an average 68 ± 8% FE over 35 h of operation with multiple on/off cycles. In situ Raman and time-dependent X-ray diffraction measurements identified metallic Sn as electrocatalytic active sites during long-term operation. Further evaluation in a 25 cm^2^ electrolyzer cell demonstrated impressive performance with a sustained current density of 500 mA cm_geo_^−2^ and an average 75 ± 6% formate FE over 24 h of operation. Our results provide additional design concepts for boosting the performance of formate-producing catalysts.

## Introduction

Electrochemical reduction of CO_2_ (CO_2_RR) powered by renewable energy is an appealing approach to produce carbon–neutral chemical feedstocks and fuels. Formic acid (HCOOH), often electrochemically produced as formate (HCOO^-^), is an attractive CO_2_RR product due to its wide uses in agricultural, chemical and pharmaceutical industries^1–4^. Formic acid/formate has also been identified as an emerging fuel for fuel cells^5,6^, a liquid hydrogen carrier with high volumetric capacity (53 g of H_2_ per liter)^7,8^, and for biomass upgrading applications^9^. Industrial formic acid production from fossil fuel precursors is extremely carbon intensive^2^, but electrochemically converting CO_2_ to formate, followed by down-stream electrodialysis purification into formic acid^10^, could provide a carbon neutral or carbon negative route for producing this versatile chemical.

Sn-based materials are some of the most effective CO_2_RR electrocatalysts for formic acid/formate production^11–18^. However, the performance of most Sn-based catalysts is still inadequate for practical applications because of low current densities (typically 10 ~ 25 mA cm_geo_^−2^ in aqueous H-cells; Table S1), high overpotentials, and poor long-term stability^11–24^. Therefore, further catalyst design efforts and demonstration in full-cell electrolyzer devices are required to boost CO_2_-to-formate conversion, improve efficiency, and validate high current density operation in realistic device architectures.

The CO_2_RR has a rich structure sensitivity, and substantial efforts have been devoted to improving performance by controlling the catalyst morphology, dimension, size, composition, crystallographic orientation, surface structure or defects^11,25–28^. For example, incorporating a second metal such as Cu, Pd, or Ni into Sn can tune the CO_2_RR selectivity of Sn to CO with 80–90% Faradaic efficiency (FE)^29–31^, while In, Bi, and Pd can strongly improve both formic acid selectivity and current density at lower overpotential^32–35^. Controlling the nanoscale surface structure could also tune the proportion of low-coordinated corner, edge, and terrace sites in the catalysts, which strongly impact the adsorption and activation of CO_2_, as well as the formation of key intermediates^19,36,37^. Three-dimensionality (3D) is another CO_2_RR electrocatalyst design consideration, and reports have described CO_2_RR catalyst morphologies assembled from nanoscale building blocks, including spheres, flowers, sheets, dendrites, porous foams, inverse opals, and others^12–18,30,35,38–43^. These 3D structures can offer larger surface area and a high density of electrocatalytic active sites that can improve current density^12–16,27,35,39,40,42^. From this perspective, a high-performance SnO_2_ electrocatalysts can be designed by combining the concepts of 3D morphology, surface structure, and size control to improve current density and formate selectivity.

Here we demonstrate a template-based synthetic approach to create hollow nanosphere catalysts constructed from SnO_2_ nanocrystal building blocks. The crystallinity and size of the constituent SnO_2_ nanocrystals were controlled by varying the calcination temperature and exhibited strong impact to the catalysts’ formate selectivity and partial current density. The best-in-class nanospheres produced 71–81% formate Faradaic efficiency (FE) in a broad potential range and achieved a maximum formate partial current density of 73 ± 2 mA cm_geo_^−2^ at −1.3 V vs. RHE. The SnO_2_ nanospheres also outperformed non-structured SnO_2_ nanoparticles (nps) and commercially available SnO_2_ nps catalysts. In situ Raman spectroscopy and time-dependent synchrotron-based X-ray diffraction (XRD) tracked the electrochemical reduction of SnO_2_ nanospheres under steady-state CO_2_RR conditions. The CO_2_RR evaluation in a 25 cm^2^ membrane electrode assembly (MEA) electrolyzer cell demonstrated sustained operation for 24 h at an industrially-relevant current density of 500 mA cm_geo_^−2^. Our results provide new design concepts for boosting the performance of formate-producing catalysts by controlling the surface structure to increase electrochemically-accessible surface area.

## Methods

### Synthesis of hierarchical hollow SnO_2_ spheres

All chemicals were purchased from Sigma-Aldrich and used as received without further purification. Hollow SnO_2_ spheres were synthesized by a combined sol–gel and templating method. Spherical PMMA template particles with diameters of ~ 210 nm were prepared by surfactant-free emulsion polymerization using a cationic free radical initiator as described in the Supplementary Information. In a typical procedure, 226 mg of tin (II) chloride dihydrate (SnCl_2_.2H_2_O) were dissolved in 5 mL of ethanol (C_2_H_5_OH, 200 proof) and 38 mg of anhydrous citric acid (C_6_H_8_O_7_) were separately mixed in 5 mL of ethanol. The citric solution was then added into the tin precursor and sonicated for 15 min. 1.5 mL of tin-citric solute ion was added dropwise into 30 mL of aqueous PMMA latex template (0.5 wt%) under vigorous stirring at room temperature. After 30 min of stirring, the mixture was evaporated overnight in the oven at 60 °C to obtain the as-synthesized powders. The same stock tin-citric solution was used to make multiple batches of as-synthesized materials which were subsequently annealed in static air at 300, 400, 500 and 600 °C for 3 h with ramping rate of 1 °C min^−1^. The obtained powder was denoted as “SnO_2_ nanospheres”.

Non-templated SnO_2_ nps were prepared using the same procedure, except the 30 ml of deionized water (DIW) did not contain the PMMA dispersion. After evaporation at 60 °C, the as-synthesized sample was subsequently calcined in air at 500 °C with a ramping rate of 1 °C min^−1^ for 3 h and named “non-templated SnO_2_ nps”. Commercial SnO_2_ nanopowder with a heterogeneous particle size distribution between 5 and 150 nm (Sigma) was also used as reference material and denoted as “com-SnO_2_ nps”.

### Electrochemical CO_2_ reduction in an H-cell

Electrochemical experiments were performed in a gas-tight, two-compartment H-cell separated by a Nafion 117 proton exchange membrane which was described in previous work^40^. Each compartment was filled with 60 mL of aqueous 0.1 M KHCO_3_ electrolyte (99.99%, Sigma-Aldrich) and contained 90 mL headspace. Ultra-pure DIW with 18.3 MΩ cm^−1^ resistivity (Barnstead EASYpure LF) was used in all electrochemical experiments. The catholyte was continuously bubbled with CO_2_ (99.999%, Butler gas) at a flow rate of 20 mL min^−1^ (pH ~ 6.8) under vigorous stirring during the experiments^40^. The counter and reference electrodes were Pt mesh and Ag/AgCl (saturated NaCl, BASi®), respectively. The catalyst ink was composed of 2.8 mg of the powder catalysts, 0.32 mg Vulcan VC-X72 carbon black, and 40 μL of Nafion® 117 solution binder (Sigma-Aldrich, 5%) in 400 μL of methanol. Working electrodes were fabricated by drop-casting the ink onto PTFE-coated carbon paper (Toray paper 060, Alfa Aesar) and N_2_-dried. The mass loadings were kept at 9.5 ± 0.6 mg_ink_ cm_geo_^−2^ (corresponding to 5.4 ± 0.3 mg_SnO2_ cm_geo_^−2^). All potentials were referenced against the reversible hydrogen electrode (RHE) (unless otherwise specified), and the uncompensated ohmic resistance was automatically corrected at 85% (*i*R-correction) using the instrument software in all electrochemical experiments^40^.

CO_2_ electroreduction tests were performed at room temperature using a SP-300 potentiostat (BioLogic Science Instrument). Short-term chronoamperometric experiments were conducted for 20 min at each applied potential between −0.6 V and −1.3 V vs. RHE and the products were collected every 20 min. Each data point is an average of at least three independent experiments on different fresh electrodes. Long-term chronoamperometric experiments were conducted over several days at −1.2 V vs. RHE and the testing was run for 5 h per day. After each day, the electrodes were discarded from the electrolyte, rinsed with DIW, and stored under ambient conditions in polystyrene petri dish. Fresh aqueous KHCO_3_ catholyte was used for each cycle. The total and partial current densities were normalized to the exposed geometric area of the catalyst (unless otherwise specified).

The evolved gas products were collected in Tedlar gas-tight bags (Supelco) and then quantified by PerkinElmer Clarus 600 gas chromatography equipped with both FID and TCD detectors, using a ShinCarbon ST 80/100 Column and He carrier gas. The liquid products collected from the catholytes were filtered with a 0.22 μm PES filter and determined by Dionex ICS-5000 + ion chromatography using ED50 conductometric detector, ASRS suppressor in auto-generation mode, AS11-HC column and gradient KOH eluent. The calculation of Faradaic efficiency (FE) for all products and formation selectivity is described in the Supplementary Information.

### Electrochemical CO_2_ reduction measurement in full MEA electrolyzer

MEA full cell characterization was performed using custom-built hardware with an active area of 25 cm^2^ previously reported in Chen et al.’s work^44^. The anode was a 25 cm^2^ Ni foam (MFNi16m, MTI Corporation) with a thickness of 1.6 mm that was placed against a triple serpentine flow channel. Polytetrafluoroethylene (PTFE) gaskets with a thickness of 1.55 mm were used to achieve an anode electrode compression of 91%.

A commercial bipolar membrane (BPM) (FBM, Fumatech GmbH, Fuel Cell Store) was used with the cation exchange layer (crosslinked poly-ether ketone) facing the cathode, and the anion exchange layer (polysulfone with bicyclic amines) facing the anode. The catholyte flow channel had a thickness of 1.27 mm and was designed as a serpentine shape with four evenly spaced fingers, with each finger having a width of 2 mm. The cathode GDE was prepared by painting SnO_2_ catalyst ink onto a gas diffusion layer (Sigracet 39BB GDL, Fuel Cell Store). Vulcan XC72R carbon powder was added to the ink to obtain a catalyst to carbon weight ratio of 1:1. A 5 wt% Nafion ionomer (D521 Nafion Dispersion, 1100 EW, Fuel Cell Store) was added to the ink with an ionomer to carbon weight ratio of 0.6:1. The fabricated GDE had a SnO_2_ loading of 0.5 mg cm^−2^. The cathode PTFE gasket was chosen to achieve a GDE compression of 18%.

The cell was heated to 60 °C during experiments and humidified CO_2_ gas was supplied to the cathode at a relative humidity of 100% and a gas flow rate of 2 SLPM. The flow rates of 0.4 M K_2_SO_4_ catholyte and 1 M KOH anolyte were 40 ml min^−1^ and 50 ml min^−1^, respectively. The liquid samples were filtered with a 0.22 µm PTFE syringe filter and then analyzed using Agilent 1260 Infinity II Bio-inert high-performance liquid chromatography. The gas products were collected with multi-layer foil gas sampling bags (Supel^l^™, Sigma-Aldrich) and analyzed using Agilent 4900 Micro gas chromatography.

### Materials characterizations

Scanning electron microscopy (SEM) imaging was performed on a FEI Quanta 600F microscope operated at 10–20 kV equipped with an energy-dispersive X-ray (EDX) detector. High-resolution transmission electron microscopy (HR-TEM) was carried out on a FEI Titan Themis G2 200 Probe Cs Corrected Scanning Transmission Electron Microscope operated at accelerating voltage of 200 kV. The powder sample was suspended in ethanol, drop-casted onto a holey carbon-supported Cu grid, and naturally dried in air. TEM images of the post-reaction sample were prepared by scratching the electrode catalyst from carbon paper, sonicating with ethanol, and then dropping onto the TEM grid. X-ray powder diffraction (XRD) patterns were collected on a PANalytical X’Pert Pro X-ray diffractometer using CuKα radiation (λ = 1.5418 Å) at a scan rate of 0.2°min^−1^.

Synchrotron X-ray diffraction measurements were conducted at beamline 17-BM-B (λ = 0.24121 Å) of the Advanced Photon Source at Argonne National Laboratory. Individual SnO_2_ nanosphere electrodes were held at −1.2 V vs. RHE for fixed amounts of time in the H-cell (e.g. 0.5 h, 1 h, 5 h, 10 h, 20 h and 30 h). These post-reaction electrodes were then removed from the H-Cell after testing for a fixed amount of time, rinsed with DIW, immediately dried under flowing N_2_, and wrapped with kapton tape to minimize air exposure. However, we do acknowledge that some reoxidation of the catalyst occurred between electrochemical testing and synchrotron XRD measurement. Two-dimensional diffraction patterns were collected by a Perkin Elmer amorphous silicon detector, data acquisition was performed with QXRD and the diffraction ring was integrated using GSAS-II freeware package^45^.

## Results and discussion

### Materials characterizations

**Figure 1:**
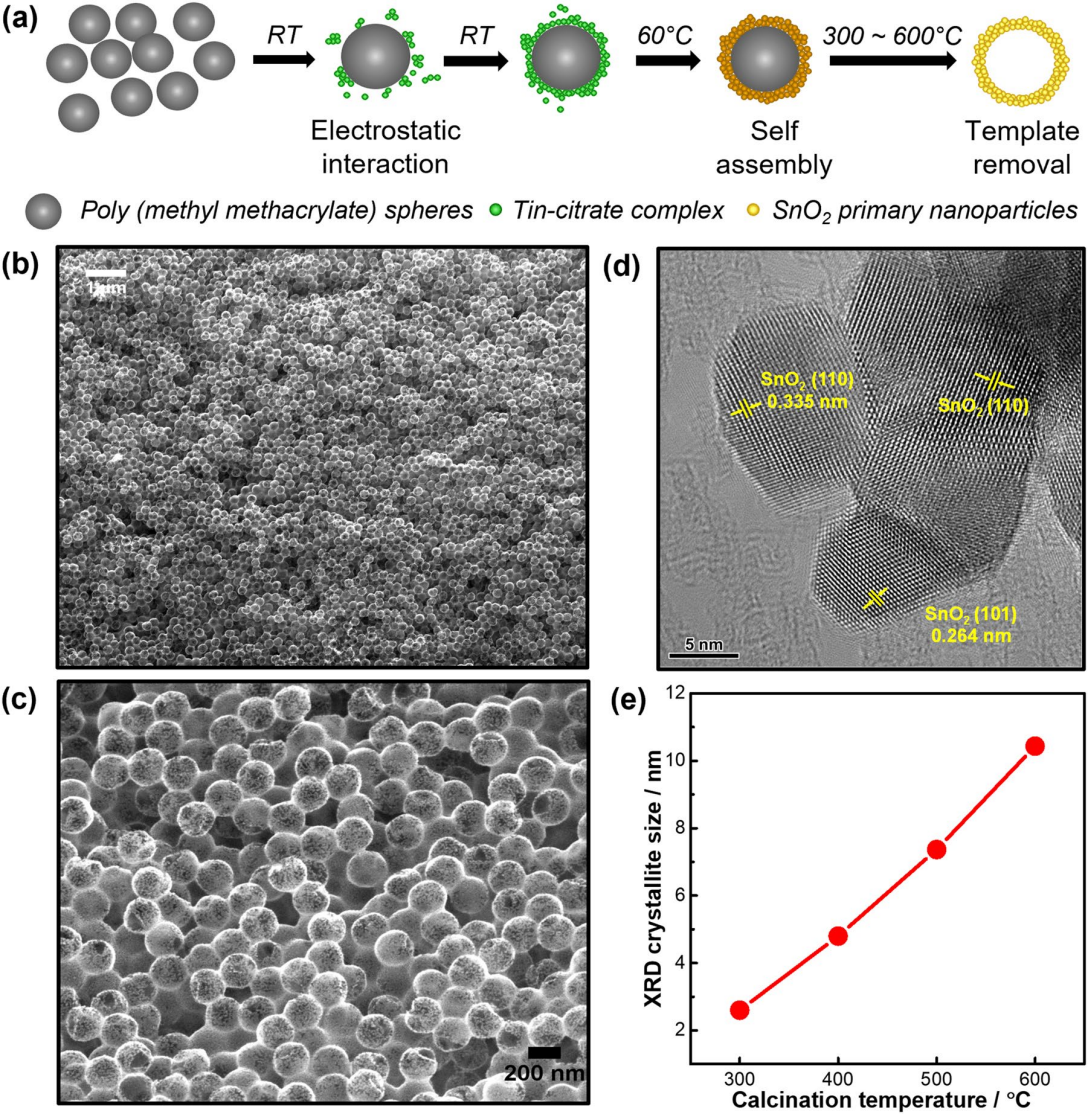
(**a**) Scheme illustrating the synthesis of 3D hollow SnO_2_ nanospheres by a combined sol–gel and templating method. (**b**,**c**) Representative FE-SEM and (**d**) HR-TEM images of SnO_2_ nanosphere calcined at 500 °C. (**e**) XRD crystallite size as function of calcination temperature.

The 3D SnO_2_ hollow nanospheres were prepared by a combined sol–gel and templating approach (Fig. 1a). Negatively charged tin (II) citrate complex was absorbed on the surface of positively charged poly (methyl methacrylate) (PMMA) spheres (diameter of *ca.* 220 nm, Fig. S1) through electrostatic interaction. The system underwent hydrolysis, condensation, nucleation, and self-assembly to create tin-containing coating layers on the surface of the PMMA spheres. Subsequent calcination in air between 300 to 600 °C not only removed the PMMA template but also converted these coating layers into SnO_2_ nanocrystals to produce hollow SnO_2_ nanospheres (Fig. 1b and Figs. S2-S4).

A representative scanning electron microscope (SEM) image in Fig. 1b,c shows a SnO_2_ nanosphere sample calcined at 500 °C having diameter of 205–210 nm. HR-TEM micrographs in Fig. 1d and Fig. S4 indicate the nanosphere walls were constructed from small, interconnected SnO_2_ nanoparticles with sizes of 6–10 nm. The PMMA template fixed almost same diameter of nanospheres for all calcination temperatures, and XRD, EXAFS and XPS results (Fig. S5) further confirm tetragonal rutile structure (space group: P4_2_/*mnm*, JCPDS 41–1445) and consistent Sn^4+^ oxidation state in all samples. Higher calcination temperatures produced sharper, more intense XRD peaks that indicate improved crystallinity and larger mean crystallite size. Figure 1e shows that the crystallite size increased from 2.5 nm to 10.5 nm when increasing the temperature from 300 °C to 600 °C. It is expected that the crystallinity and crystallite size of 3D SnO_2_ nanospheres would impact the CO_2_RR activity of SnO_2_ nanospheres.

### Electrochemical CO_2_ reduction performance in conventional H-cell

**Figure 2:**
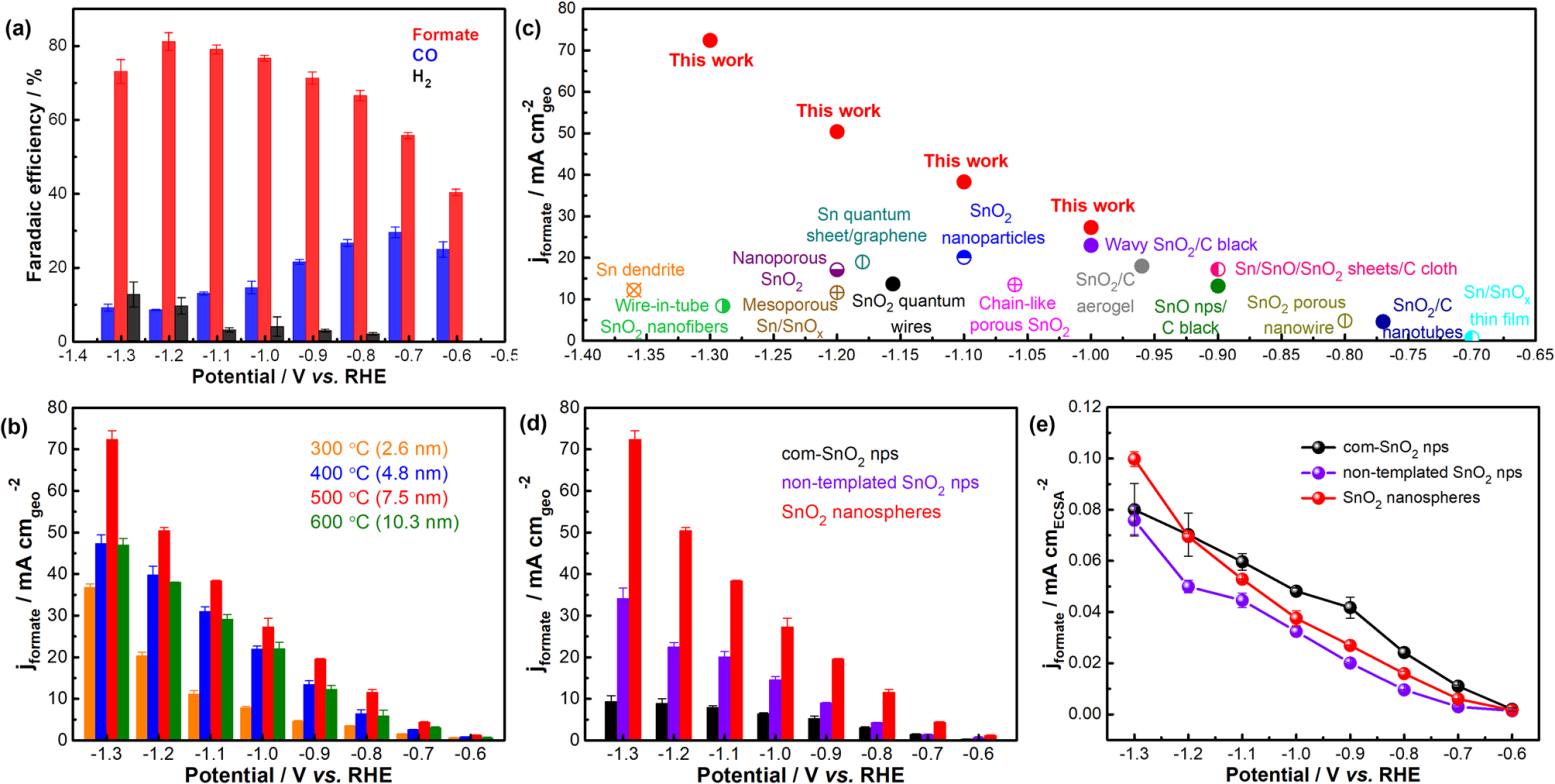
(**a**) Representative Faradaic efficiency for formate, CO, and H_2_ vs. cathodic potentials for SnO_2_ nanospheres calcined at 500 °C. (**b**) Potential-dependent formate partial current density as function of calcination temperature of SnO_2_ nanospheres (aka SnO_2_ crystallite size). (**c**) Comparison of CO_2_RR performance for the best-performing SnO_2_ nanospheres with previously reported Sn, SnO_2_ and SnO_2_-carbon electrocatalysts tested in a H-cell with bicarbonate electrolyte (mixed metal oxides, alloys, and doped systems are excluded): Sn dendrite^12^, nanoporous SnO_2_^15^, SnO_2_ porous nanowires^16^, chainlike mesoporous SnO_2_^18^, Sn/SnO_x_ thin film^20^, Sn/SnO/SnO_2_ nanosheets/carbon cloth^21^, wire-in-tube SnO_2_ nanofibers^22^, SnO_2_ nanoparticles^23^, ultrathin SnO_2_ quantum wires^24^, SnO_2_/carbon nanotubes^46^, Sn quantum sheet/graphene^47^, SnO_2_/carbon aerogel^48^, SnO nanoparticles/carbon black^49^, mesoporous Sn/SnO_x_^50^, wavy SnO_2_/carbon black^51^. (**d**,**e**) Comparison of (**d**) geometric formate partial current density and (**e**) ECSA-normalized formate current density for commercially-available SnO_2_ nps (com-SnO_2_ nps), non-templated SnO_2_ nps, and the best-performing SnO_2_ nanospheres calcined at 500 °C.

The CO_2_RR performance of nanosphere catalysts was screened between −0.6 V and −1.3 V vs. RHE in a H-cell containing CO_2_-saturated 0.1 M KHCO_3_. Figure 2a shows representative FEs for formate, CO, and H_2_ products vs. applied potentials for SnO_2_ nanospheres calcined at 500 °C which were built from ~ 7.5 nm crystallite size. This nanosphere catalyst sample produced 71–81% formate FE between −0.9 V and −1.3 V vs. RHE, and the FE for C_1_ products (formate and CO) reached > 90% between −0.8 V to −1.2 V vs. RHE (Fig. S6). SnO_2_ nanospheres calcined at other temperatures also produced formate as a main product (Fig. S7), albeit with lower FEs than the 500 °C nanospheres, along with smaller amounts of CO and H_2_. It is worth mentioning that gaseous CO and H_2_ side-products (syngas) are easily separated from liquid formate for subsequent use in methanol or Fischer–Tropsch synthesis.

Figure 2b compares the formate partial current densities of SnO_2_ nanospheres calcined at different temperatures, and 500 °C SnO_2_ nanospheres produced the highest formate partial current density at all potentials (maximum value of 73 ± 2 mA cm_geo_^−2^ at −1.3 V vs. RHE). This result implies an apparent dependency between CO_2_RR performance and the size of the constituent SnO_2_ nanocrystals. For example, SnO_2_ nanospheres annealed at 300 °C contained the smallest SnO_2_ nanocrystals (~ 3 nm) and produced the highest H_2_ FE. This observation is also consistent with previous reports for increased H_2_ production from small Sn, Cu, and Au nps and points to undercoordinated surface sites as likely H_2_ evolution centers^19,29,52,53^. Higher calcination temperatures, *i.e.,* 400 and 500 °C, produced ~ 5 nm and ~ 7.5 nm SnO_2_ nanocrystals, resulting in higher formate current densities and reduced H_2_ production. Calcination at 600 °C further increased the nanocrystal diameter to > 10 nm and decreased formate production, which is qualitatively consistent with previous size-dependent results for SnO_2_ catalysts^19^. Therefore, we suggest that SnO_2_ nanospheres annealed at 500 °C likely produced an optimum balance between crystallinity and nanocrystal size, and thus maximized formate selectivity and partial current density. In fact, the data summarized in Fig. 2c and Table S1 demonstrate the 500 °C SnO_2_ nanospheres produced some of the highest formate partial current densities ever reported for Sn, SnO_2_ and SnO_2_-carbon electrocatalysts in aqueous H-Cells^12,15,16,18,20–24,46–51^.

We also compared the performance of non-templated, ~ 7 nm SnO_2_ nps and commercially-available SnO_2_ nanoparticles (named com-SnO_2_ nps) with a heterogeneous size distribution between a few nanometers to hundreds of nanometers (Figs. S8 and S9). Figure 2d and Fig. S10 show a two ~ sixfold improvement in formate partial current density, 20–30% higher formate FE, and reduced H_2_ evolution for the SnO_2_ nanospheres compared with the non-templated and commercial SnO_2_ nps. Impressively, total FE for the C_1_ products for the 500 °C SnO_2_ nanospheres was substantially higher than either non-templated or commercially available SnO_2_ catalysts between −0.6 V and −1.3 V vs. RHE (Fig. S10).

We attribute the higher CO_2_RR performance of SnO_2_ nanospheres to a larger surface area, as confirmed with both BET and capacitance-based electrochemical surface area (ECSA) measurements^13,18,24,42^ (Fig. S9, S11 and Table S2). In particular, the 500 °C nanospheres demonstrated 1.5–3 times larger ECSA than non-templated and commercially-available SnO_2_ nanoparticles. These results confirm the nanosphere catalysts contained a higher density of electrocatalytic active sites to participate to CO_2_RR. As shown in Fig. 2e, all three samples produced comparable ECSA-normalized formate partial current density, indicating that the total amount of electrochemically-active surface area was the dominant influence on *geometric* formate partial current density. In this regard, controlling the crystallinity and surface structure to maximize ECSA is a viable route for improving geometric current density.

**Figure 3:**
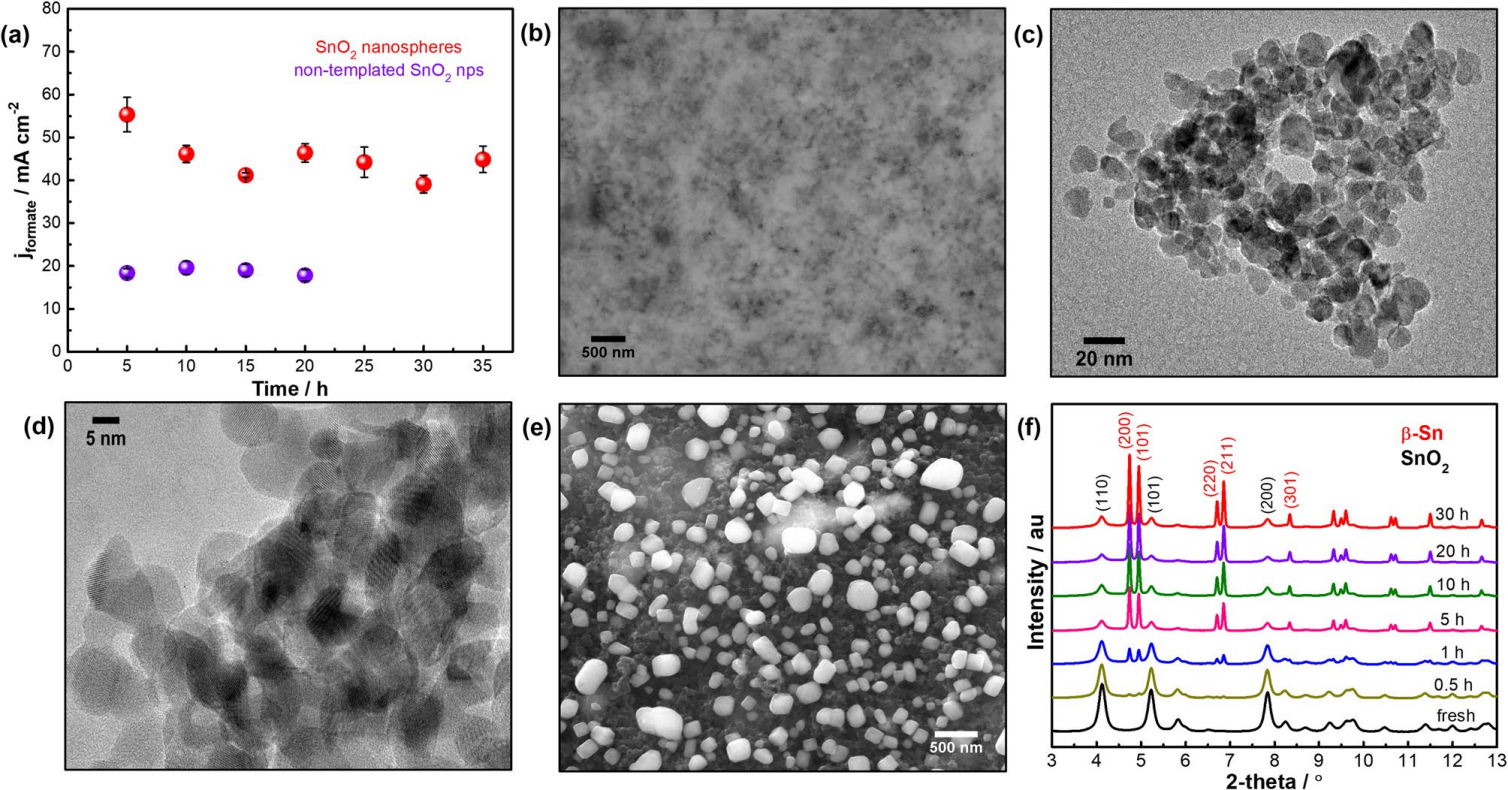
(**a**) Long-term CO_2_RR performance of the best performing SnO_2_ nanospheres and non-templated SnO_2_ nps at −1.2 V vs. RHE. The experiments were run intermittently over multiple 5-h electrolysis periods. (**b**) Back-scattered SEM and (**c**,**d**) TEM images of SnO_2_ nanospheres electrode after 35 h of operation. (**e**) SEM image of non-templated SnO_2_ nps after 20-h electrolysis. (**f**) Time-dependent synchrotron-based XRD profiles of SnO_2_ nanospheres collected at −1.2 V vs. RHE.

The long-term electrolysis of SnO_2_ nanospheres catalyst was conducted at −1.2 V vs. RHE in an H-cell over multiple start/stop cycles (Fig. 3a and Fig. S12). This potential produced the highest formate FE during short-term electrolysis experiments and on/off cycles mimicked operating with intermittent renewable electricity. The time-dependent formate partial current density is shown in Fig. 3a and the time-dependent FEs for formate, CO and H_2_ are shown in Fig. S12. The catalyst showed a small initial decline in formate partial current density from ~ 55 mA cm_geo_^−2^ in the first 5 h to an average 45 ± 5 mA cm_geo_^−2^ over the next 30 h of operation, and we observed an average 68 ± 8% formate FE during the entire long-term experiment. Post-electrolysis electron microscopy in Fig. 3b–d revealed the SnO_2_ nanocrystal size increased from ~ 7.5 nm to 15–25 nm. Conversely, as shown in Fig. 3a, non-templated SnO_2_ nanoparticles produced much lower formate partial current density (~ 20 mA cm_geo_^−2^) and experienced severe particle agglomeration after 20 h (Fig. 3e). Similar particle size growth was also observed previously for other SnO_2_ nanopowder electrocatalyts^18,39,54^. While the initial 3D nanosphere structure reconstructed during extended electrolysis, our results show the initial spherical structure prevented large-scale growth of the constituent SnO_2_ nanocrystals, whereas non-templated SnO_2_ nanoparticles experienced dramatic particle agglomeration.

We conducted time-dependent, synchrotron-based XRD measurements to gain insight into the structural evolution of SnO_2_ nanospheres during long-term electrolysis at −1.2 V vs. RHE. Figure 3f revealed the SnO_2_ nanocrystals reduced into metallic Sn as evidenced by the emergence of body-centered tetragonal β-Sn diffraction peaks (space group: I4_1_/*amd*). These results also indicate rapid transformation of SnO_2_ into metallic Sn and an increase in crystallite size to *ca.* 23 nm under steady state operation (Fig. S13). Notably, this crystallite size remained stable over 30 h of operation and the XRD data agrees well with the post-electrolysis imaging in Fig. 3b–d that ruled out severe particle growth during long-term electrolysis. We also observed a minor residual oxide phase that likely resulted from reoxidation upon air exposure between electrochemical testing and XRD measurement.

Despite the literature containing multiple reports on in situ X-ray absorption, Raman and IR spectroscopies for SnO_2_ CO_2_RR catalysts^35,55–57^, the results in Fig. 3f represent the first time-dependent XRD study to successfully track the phase change of SnO_2_ catalysts as a function of CO_2_RR electrolysis time. Despite some reoxidation between electrochemical testing and ex situ XRD measurements, our XRD results suggest the catalyst was reduced during CO_2_RR at −1.2 V vs. RHE. This data strongly supports complementary in situ Raman spectroscopy experiments that showed the attenuation and then complete disappearance of SnO_2_ characteristic bands at −1.2 V vs. RHE (Fig. S14). This result provides further evidence for the fast reduction of the SnO_2_ surface to metallic Sn during CO_2_RR and our observation is consistent with previous *operando* Raman results for other SnO_2_ electrocatalysts^55,56^. Combined bulk and surface techniques in the present provide strong evidence that metallic Sn species are indeed the electrocatalytic active sites for converting CO_2_ into formate.

### CO_2_ electroreduction to formate in high-performance membrane electrolyte assembly (MEA) electrolyzer

Practical CO_2_ conversion applications will require operating in electrolyzer devices at current densities of ~ 100 s mA cm^−2^^58^. Consequently, we evaluated the CO_2_RR performance of SnO_2_ nanosphere catalysts on 25 cm^2^ gas diffusion electrodes (GDEs) in a recently reported MEA electrolyzer cell (details in the Methods Section)^44^. A thin aqueous catholyte layer between bipolar membrane (BPM) and cathode GDE reduced proton concentrations near the cathode interface, suppressed H_2_ production, and transported away liquid products. This type of MEA full cell design can produce much higher current density than H-cells because gaseous CO_2_ is delivered directly to the cathode, rather than converting CO_2_ dissolved in aqueous electrolyte, and it allows catalyst evaluation under conditions that are more representative of scalable device architectures.

**Figure 4:**
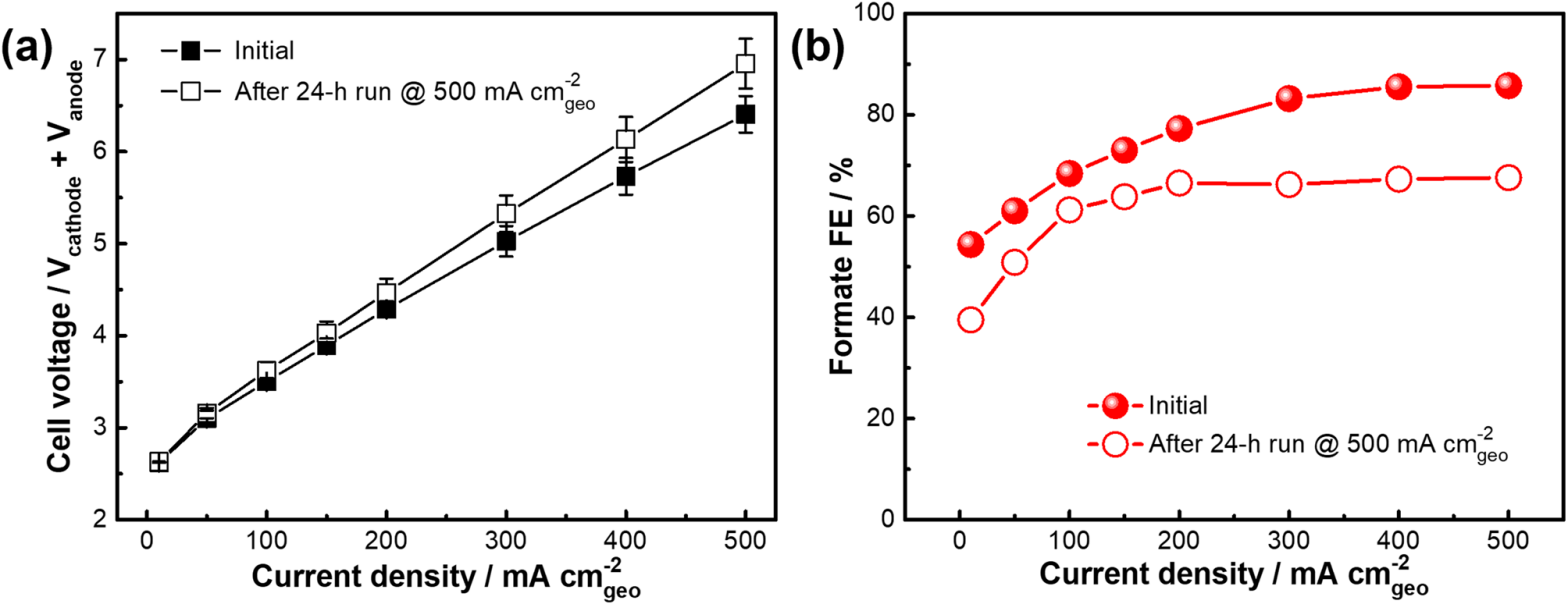
(**a**) MEA full cell polarization curves collected before and after a 24-h electrolysis at 500 mA cm_geo_^−2^. (**b**) Corresponding formate FEs vs. geometric current density. The MEA full cell contained a 5 cm × 5 cm cathode GDE decorated with SnO_2_ nanospheres, a Ni foam anode, and a bipolar membrane with aqueous 0.4 M K_2_SO_4_ catholyte and aqueous 1 M KOH anolyte.

Figure 4a shows the initial polarization curve of the MEA cell operated at geometric current densities of 50–500 mA cm_geo_^−2^. The cell voltage and FE for formate increased with increasing current density and the cell reached current density of 500 mA cm_geo_^−2^ at 6.4 V with 86% formate FE (Fig. 4b). An equilibrium cell potential of 1.41 V is required for the CO_2_ electrolysis to produce formic acid, however, due to the simultaneous formation of CO, the optimum voltage is above 3V^58^. As previously shown^44^, approximately 43% of the cell voltage contribution stems from the BPM (junction and associated ion exchange layers), with 15% and 23% of the voltage contribution coming from catholyte layer and the cathode overpotential, respectively. The cell was then held at a constant 500 mA cm^−2^ for 24 h and demonstrated an average 75 ± 6% formate FE (Fig. S15) and energy efficiencies between 14–19% (Fig. S16). We did observe a gradual increase in cell voltage over the 24-h test that was primarily associated with BPM degradation and oxidation of the Ni foam anode (Fig. S17). No nickel contamination from the anode was detected on the cathode GDE after the 24-h electrolysis (Fig. S18–S19). The BPM and anode were replaced, and the final polarization curve achieved 500 mA cm^−2^ current density and 68% formate FE (Fig. 4b), albeit at an approximately 9% higher cell voltage than the initial polarization. As summarized in Table S3, the sustained CO_2_ conversion at 500 mA cm_geo_^−2^ using a 25 cm^2^ active electrode area in the present study is among the highest reported in the literature for pure SnO_2_ catalysts in MEA electrolyzer cells^44,59,60^. The loss of formate FE over extended time run in an electrolyzer for Sn-based catalysts has been reported^58–60^, however the opportunities still exist to further improve system performance through component-level optimization of the BPM^61^, electrode and cell architecture, GDE transport property deterioration and flooding^62^, and operational parameters.

## Conclusions

We have reported SnO_2_ nanosphere electrocatalysts constructed from small, interconnected SnO_2_ nanocrystals for highly efficient CO_2_ conversion into formate. Tuning thermal annealing temperatures maximized formate production by optimizing the crystallinity and particle size of the constituent SnO_2_ nanoparticles. The best performing SnO_2_ nanospheres demonstrated high Faradaic efficiencies, selectivities, and superior current densities toward formate production over a wide potential range in H-cell testing. SnO_2_ nanospheres surpassed non-templated SnO_2_ nps of similar size and commercially-available SnO_2_ catalysts, which we attributed to larger electrochemical surface area. Finally, evaluation in a high-performance MEA full cell electrolyzer device demonstrated the SnO_2_ nanospheres could sustain impressive CO_2_ conversion into formate over 24-h at an industrially-relevant current density of 500 mA cm_geo_^−2^. Our work demonstrates the utility of incorporating 3D structure into CO_2_RR electrocatalysts and provides additional catalyst design principles for improving performance.

## Supplementary Information

Supplementary Information.

## Data Availability

All data included in this study are available from the corresponding authors and can be provided upon request as needed.
