# Supplementary material for: High current density electroreduction of CO2 into formate with tin oxide nanospheres
Source: Sci Rep. 2022 May 19;12:8420. doi: 10.1038/s41598-022-11890-6 (PMC9120473; doi:10.1038/s41598-022-11890-6)
Supplement: Supplementary file 1 — Supplementary Information. [file 41598_2022_11890_MOESM1_ESM.pdf]

Supplementary Information

High Current Density Electroreduction of CO_2 into Formate with Tin Oxide Nanospheres

Thuy-Duong Nguyen-Phan,[1,2]* Leiming Hu,[3] Bret H. Howard,[1] Wenqian Xu,[4] Eli Stavitski,[5] Denis Leshchev,[5] August Rothenberger,[1] Kenneth C. Neyerlin,[3]* and Douglas R. Kauffman[1]*

[1] National Energy Technology Laboratory, 626 Cochrans Mill Road, P.O. Box 10940, Pittsburgh, PA 15236-0940, USA

[2] NETL Support Contractor, 626 Cochrans Mill Road, P.O. Box 10940, Pittsburgh, PA 15236-0940, USA

[3] National Renewable Energy Laboratory, Golden, CO 80401, USA

[4] X-ray Science Division, Advanced Photon Source, Argonne National Laboratory, Argonne, Illinois 60439, USA

[5] Photon Sciences Division, National Synchrotron Light Source II, Brookhaven National Laboratory, Upton, New York 11973, USA

Corresponding Authors:

* ThuyDuong.NguyenPhan@netl.doe.gov; Douglas.Kauffman@netl.doe.gov; Kenneth.Neyerlin@nrel.gov

1. Experimental section

1.1. Synthesis of poly (methyl methacrylate) (PMMA) latex

All chemicals were purchased from Sigma-Aldrich and used as received without further purification. PMMA latex was prepared by surfactant-free emulsion polymerization using a cationic free radical initiator. 875 mL of deionized water (DIW) and 100 g of methyl methacrylate were mixed at room temperature under a nitrogen flow for 30 min and then maintained at 70 °C. Subsequently, a solution containing 0.15 g of 2,2'-azobis (2-methylpropionamidine) dihydrochloride and 25 mL of DIW was quickly added under vigorous stirring to form a milky white suspension. The suspension was then stirred at 70 °C for 6 h to complete the polymerization. After cooling down to room temperature for 1 h, the concentration of obtained PMMA latex (size of *ca.* 220 nm) was 10 wt%. The latex was diluted with DIW to achieve 0.5 wt% for further use.

1.2. Characterizations

The thermogravimetric analysis (TGA) was conducted on a Mettler Toledo DSC/TGA 3+ under flowing air from room temperature to 550 °C at a rate of 1 °C min^{-1}. X-ray photoelectron spectroscopy (XPS) was carried out on a PHI 5000 VersaProbe III scanning XPS microprobe (Physical Electronics, ULVAC-PHI Inc) using Al Kα (1486.6 eV) radiation source and a hemispherical analyzer. All the binding energies were internally calibrated to the surface adventitious hydrocarbon feature (C 1s) at 284.6 eV. N_2 sorption analysis was measured on Quantachrome Autosorb-1 at -196 °C to obtain Brunauer–Emmett–Teller (BET) specific surface area. Powder samples were degassed at 120 °C for 2 hours prior to analysis.

In situ Raman spectroscopy was performed on a LabRam HR-Evolution spectrometer (Horiba Scientific) with a 633 nm laser as an excitation source and 50x long-working-distance objective using a custom-made electrochemical cell. The composition of catalyst ink was identical to the one used in CO_2RR H-cell tests with 5 μL of the catalyst ink drop-casted onto

a glassy carbon working electrode. A Pt wire and Ag/AgCl were used as counter and reference electrodes, and iR-correction was applied in all measurements. 5 mL of 0.1 M aqueous $KHCO_3$ electrolyte was continuously purged with CO_2 during the measurements and sequential Raman spectra were collected under open circuit and at -1.2 V *vs.* RHE.

Sn K-edge X-ray absorption spectroscopy (XAS) was collected at the 8-ID (ISS) beamline of the National Synchrotron Light Source II (NSLS-II) at Brookhaven National Laboratory using a Passivated Implanted Planar Silicon detector and Sn foil for energy calibration (29.2 keV). All synthesized SnO_2 samples, bulk SnO_2 and bulk SnO powders were loaded into Kapton capillary and Sn K-edge data were collected in fluorescence modes and subsequently analyzed using IFEFFIT freeware package [1].

1.3. Calculation of Faradaic efficiency and selectivity

- The Faradaic efficiency (FE) for product i is defined as the percentage of supplied electrons used to convert CO_2 into product i and calculated as follows:

$$FE_i = \frac{z_i * F * n_i}{I * t} = \frac{z_i * F * n_i}{Q}$$

where z_i is the number of electrons involved in the formation of product i (z = 2 for formate, CO, and H_2); F is the Faraday's constant (96485 C $mole^{-1}$); n_i is the number of moles of product i formed (determined by GC and IC); I is the total current; t is electrolysis time; and Q is total charge in Coulombs passed across the electrode.

- The formate selectivity is defined as molar ratio of formate compared with the total CO_2RR products:

$$S_{formate} = \frac{mol_{formate}}{mol_{formate} + mol_{CO}}$$

1.4. Calculation of electrochemical surface area (ECSA)

The ECSA of all the electrocatalysts was estimated from the electrochemical double layer capacitance (C_{dl}). Cyclic voltammetry was scanned in the potential range where there are no Faradaic processes occurring at various scan rates in CO_2-saturated 0.1 M $KHCO_3$ electrolyte. The preparation and mass loading on PTFE-coated Toray carbon paper of working electrodes are similar to the CO_2 reduction test in H-cell. C_{dl} value was derived from the linear slope of the capacitive current density vs. scan rate plot using the following equation:

$$ECSA = \frac{C_{dl} * S_{geo}}{C_s}$$

where C_s is specific capacitance for smooth Sn surface (C_s = 20 μF cm^{-2}) [2] and S_{geo} is the geometric area of working electrode.

1.5. Calculation of energy efficiency

The energy efficiency (%), which represents total energy utilization towards formate production in electrolyzer cell, was calculated using the following equation [3]:

$$Energy\ efficiency = \frac{\frac{FE_{formate} * I}{n * F} * \Delta G}{I * V_{cell}} * 100\%$$

where $FE_{formate}$ is Faradaic efficiency for formate; I is total current density; F is the Faraday's constant (96485 J mol^{-1} K^{-1}); n = 2 is electron number for formate formation; V_{cell} is the electrolyzer cell voltage; and ΔG = 276 kJ/mol is the Gibbs free energy change [3].

2. Supporting Tables

Table S1. Comparison of CO_2-to-formate performance of 3D hierarchical SnO_2 spheres and other Sn based electrocatalysts reported for formic acid/formate production (excludes mixed metal oxides, alloys, and doped systems).

Catalysts	Electrolyte	Potential at maximum $FE_{formate}$ [V]	Maximum $FE_{formate}$ [%]	Formate current density [mA cm_{geo}^{-2}]	References
3D SnO_2 nanospheres	0.1 M $KHCO_3$	-1.2 V *vs.* RHE	81.1	50.4	This work
	0.1 M $KHCO_3$	-1.0 V *vs.* RHE	76.6	28.9	This work
Sn/SnO_x thin film	0.5 M $NaHCO_3$	-0.7 V *vs.* RHE	42	0.7	[4]
Hierarchical Sn dendrite	0.1 M $KHCO_3$	-1.36 V *vs.* RHE	71.6	12.2	[5]
Sn plate	0.1 M $KHCO_3$	-1.8 V *vs.* Ag/AgCl	80	6.4	[6]
Coralline-structured SnO_x	0.5 M $KHCO_3$	-1.6 V *vs.* SHE	87.1	8	[7]
Nanoporous Sn foam	0.1 M $NaHCO_3$	-2.0 V *vs.* Ag/AgCl	90	20.7	[8]
Chainlike mesoporous SnO_2	0.1 M $KHCO_3$	-1.06 *vs.* RHE	82	13.5	[9]
Urchin like SnO_2 microstructure	0.5 M $KHCO_3$	-1.0 V *vs.* SHE	62	1.3	[10]
Electrochemically reduced SnO_2 porous nanowires	0.1 M $KHCO_3$	-0.8 V *vs.* RHE	80.1	4.81	[11]
SnO_2 nanoparticles	0.1 M $KHCO_3$	-1.1 V *vs.* RHE	85	20.1	[12]
Wire-in-tube structured SnO_2 nanofibers	0.1 M $KHCO_3$	-1.29 V *vs.* RHE	70	8.4	[13]
Grain boundary rich SnO_2 nanoparticles (<5 nm)	1 M KOH	-0.73 V *vs.* RHE	74	51.8	[14]
Sn rod	Pure water	-1.6 V *vs.* Ag/AgCl	94.5	0.12	[15]

Table S1 (continued). Comparison of CO_2-to-formate performance of 3D hierarchical SnO_2 spheres and other Sn based electrocatalysts reported for formic acid/formate production (excludes mixed metal oxides, alloys, and doped systems).

Catalysts	Electrolyte	Potential at maximum $FE_{formate}$ [V]	Maximum $FE_{formate}$ [%]	Formate current density [mA cm_{geo}^{-2}]	References
Ultrathin sub-2 nm SnO_2 quantum wires	0.1 M $KHCO_3$	-1.156 V *vs.* RHE	87.3	13.7	[2]
Mesoporous Sn/SnO_x	0.1 M $KHCO_3$	-1.2 V *vs.* RHE	89.6	11.6	[23]
SnO_2 nanoflakes	0.5 M $KHCO_3$	-1.0 V *vs.* RHE	82.1	10.3	[24]
Nanoporous SnO_2	0.1 M $KHCO_3$	-1.2 V *vs.* RHE	90	17.1	[25]
Mesoporous SnO_2 nanosheets	0.5 M $KHCO_3$	-1.3 V *vs.* RHE	90	7.5	[26]
Ultra-small SnO nanoparticles/carbon black	0.5 M $KHCO_3$	-0.9 V *vs.* RHE	66	13.2	[16]
SnO_2/carbon aerogels	1 M $KHCO_3$	-0.96 V *vs.* RHE	76	18	[17]
Reduced SnO_2 nanoparticles/graphene	0.1 M $NaHCO_3$	-1.8 V *vs.* SCE	93.6 (graphene)	9.5 (graphene)	[18]
Reduced SnO_2 nanoparticles/carbon black	0.1 M $NaHCO_3$	-1.8 V *vs.* SCE	86.2 (carbon black)	5.3 (carbon black)	[18]
SnO_x/MWCNTs	0.1 M $KHCO_3$	-1.4 V *vs.* SCE	64	3.2	[19]
SnO_2-CNT	0.5 M $KHCO_3$	-0.77 V *vs.* RHE	76	4.6	[20]
Mesoporous SnO_2 nanosheets/carbon cloth	0.5 M $NaHCO_3$	-1.6 V *vs.* Ag/AgCl	87	45	[21]
SnO_x nanosheets/MWCNTs	0.5 M $KHCO_3$	-1.25 V *vs.* SHE	77	6.5	[22]
Sn quantum sheets/graphene	0.1 M $KHCO_3$	-1.8 V *vs.* SCE	89	19	[27]
Wavy SnO_2/carbon black	0.1 M $KHCO_3$	-1.0 V *vs.* RHE	87.4	23	[28]
Sn/SnO/SnO_2 nanosheets/carbon cloth	0.5 M $KHCO_3$	-0.9 V *vs.* RHE	89.6	17.2	[29]

Table S2. BET surface area (S_{BET}), double-layer capacitance (C_{dl}), and electrochemical surface area (ECSA) for SnO_2 nanospheres calcined at 500 °C, non-templated SnO_2 nps, and com-SnO_2 nps. All measurements were carried out in CO_2-purged 0.1 M $KHCO_3$ and all electrodes had equivalent SnO_2 loadings of 5.4±0.3 mg_{SnO2} cm_{geo}^{-2} on Toray carbon paper (total ink loading, including SnO_2 and carbon black, was 9.5±0.6 mg_{ink} cm_{geo}^{-2}).

Sample	S_{BET} [cm^2 g^{-1}]	C_{dl} [mF cm^{-2}]	ECSA [cm^2]
SnO_2 nanospheres	45.3	14.52	51.3
Non-templated SnO_2 nps	39.9	3.24	31.8
Com-SnO_2 nps	20.0	2.67	16.8

Table S3. Summary of current density, cell voltage, and energy efficiency on Sn/SnO_2 electrocatalysts for formate/formic acid production in electrolyzer cell.

Catalyst	Catholyte	Current density [mA cm^{-2}]	Cell voltage [V]	Energy efficiency [%]	Reference
SnO_2 nanospheres	K_2SO_4	150	3.9	26.8	This work
	K_2SO_4	200	4.29	25.8	This work
	K_2SO_4	300	5.0	23.7	This work
	K_2SO_4	500	6.4 ~ 7.0	13.9 ~ 19.2	This work
Sn nanoparticles	Deionized water	140	3.3	34.9	30
Sn nanoparticles	KCl + $KHCO_3$	150	5.3	14.4	31
Sn nanoparticles	KCl + $KHCO_3$	200	4.0	19.3	32
SnO_2 nanoparticles	$KHCO_3$	200	6.0	17.9	33
	$KHCO_3$	300	8.0	6.3	33
SnO_2 nanoparticles	KCl + $KHCO_3$	300	6.2	12.3	34
SnO_2 nanoparticles	K_2SO_4	500	5.9	15.3	35

3. Supporting Figures

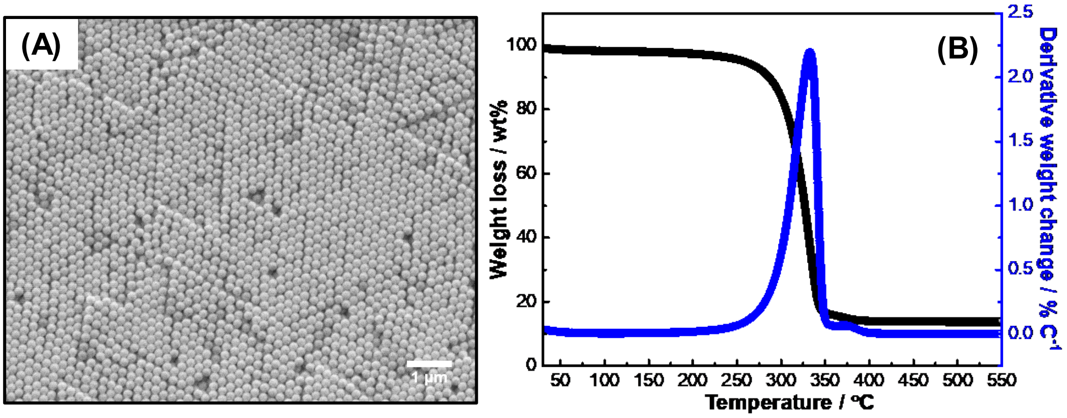

Figure S1. (A) SEM image of spherical PMMA template in diameters of ~220 nm; (B) TGA (in flowing air) and first derivative thermogravimetry (DTG) profiles of PMMA-tin-citrate as-synthesized powder obtained after self-assembly and evaporation at 60 °C, showing that the polymers were burnt off above 300 °C in air.

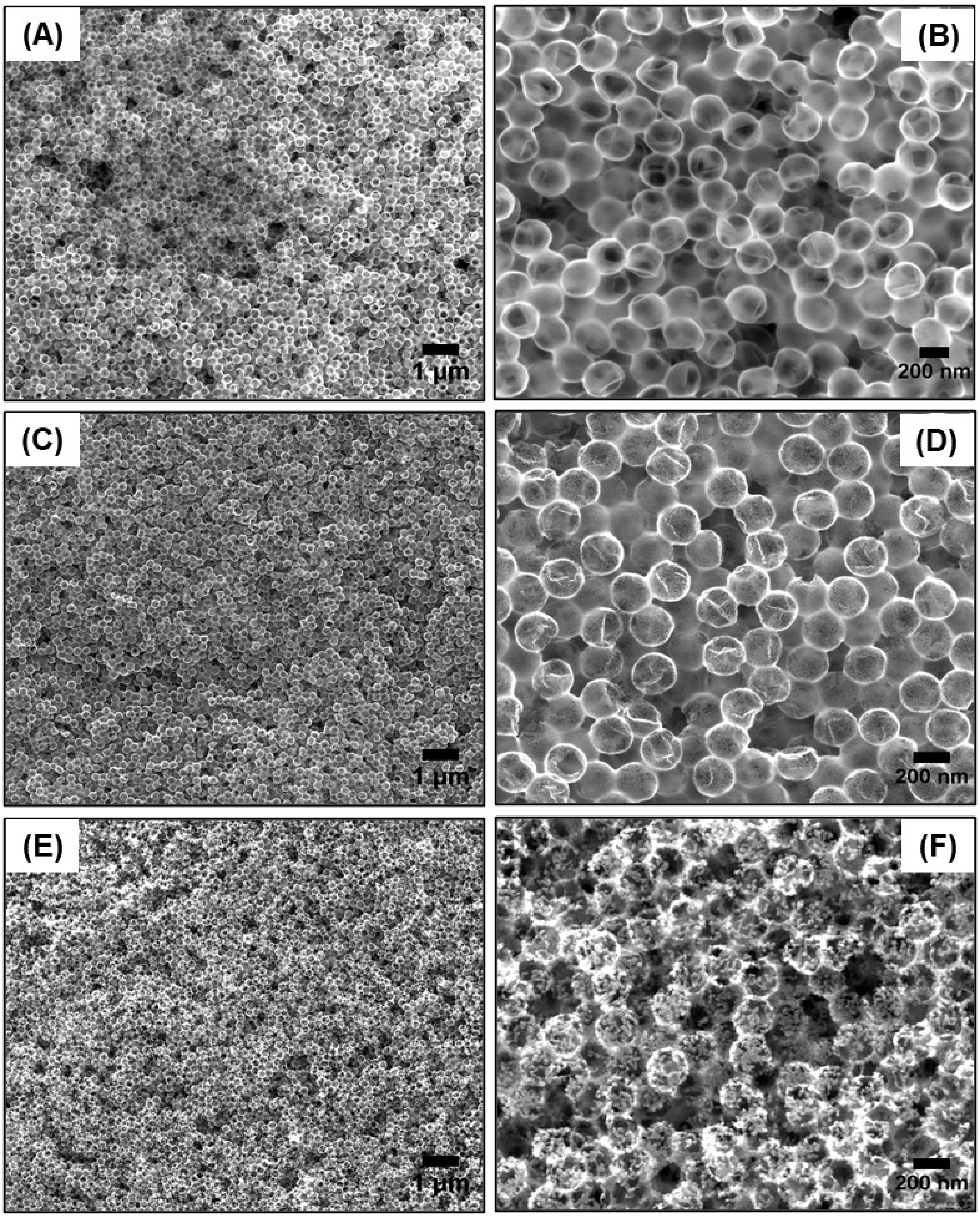

Figure S2. FE-SEM images of SnO_2 nanospheres calcined at (A, B) 300 °C, (C, D) 400 °C and (E, F) 600 °C.

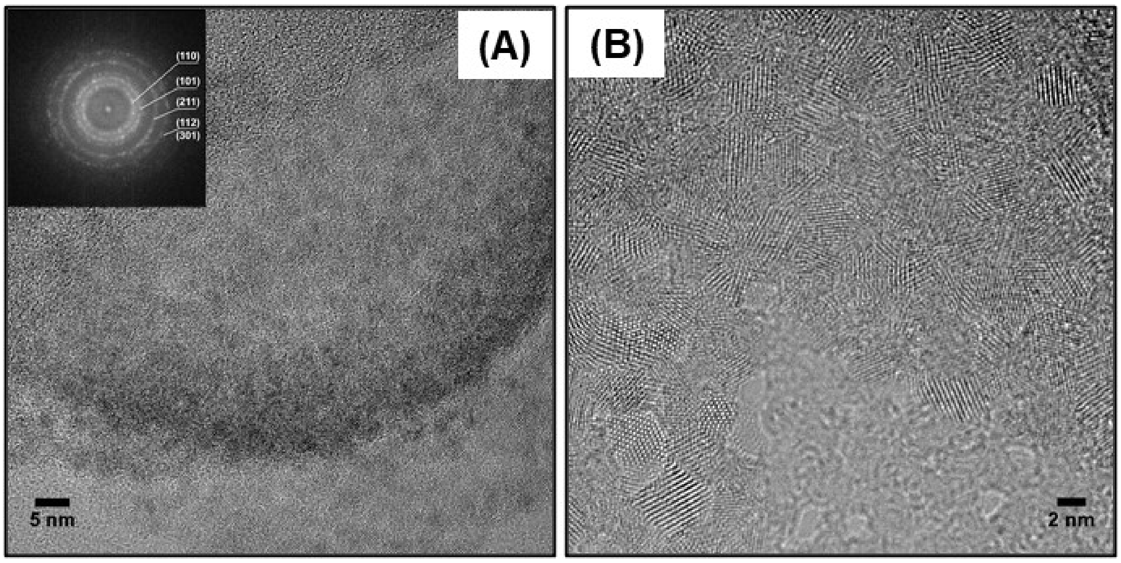

Figure S3. HR-TEM images of SnO_2 nanospheres calcined at 300 °C, revealing the spherical shells were *ca.* 5 nm-thick and composed of 2-3.5 nm SnO_2 nanocrystallites. Inset of (A) is the corresponding FFT diffraction pattern showing polycrystalline tetragonal rutile SnO_2.

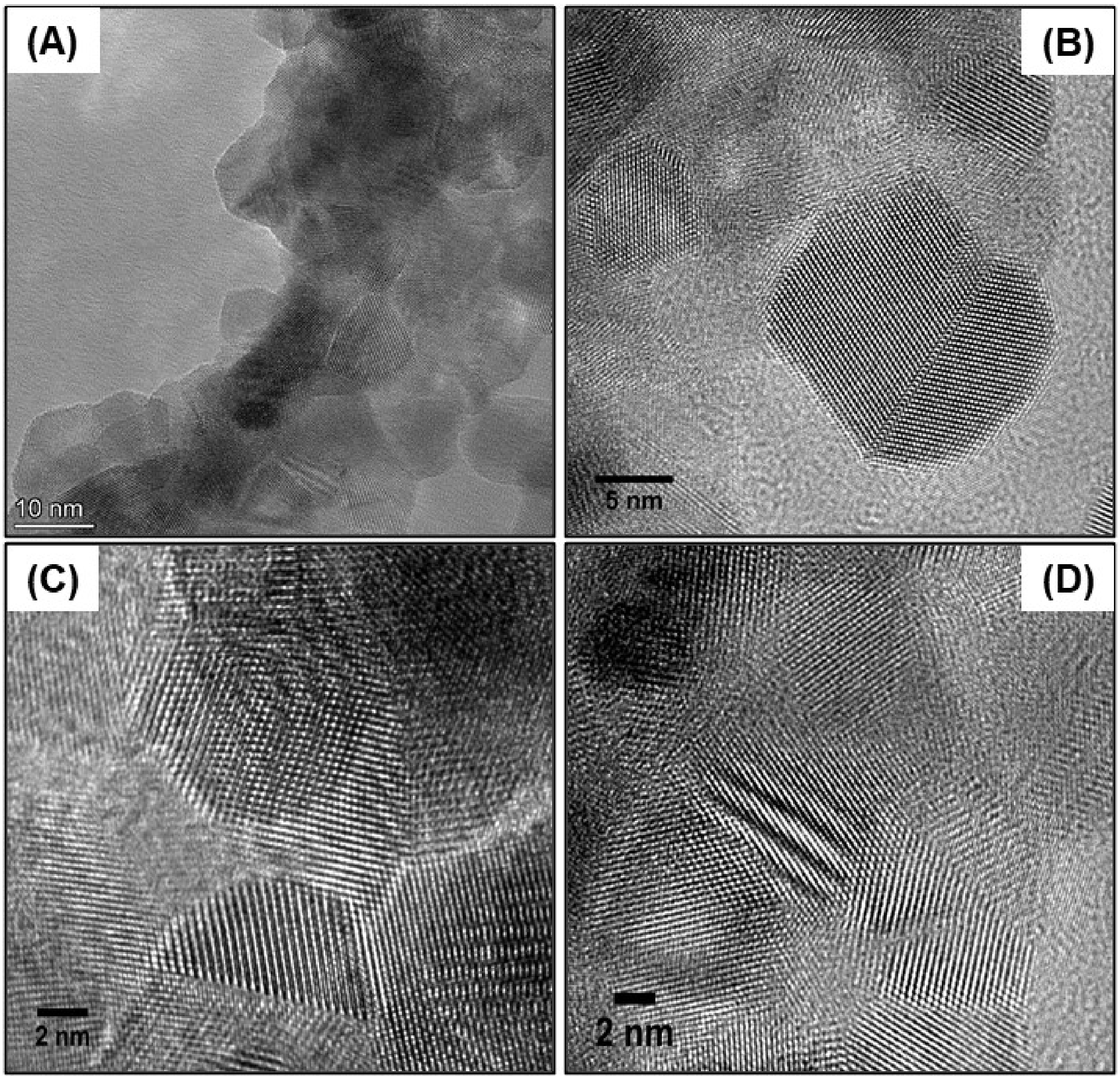

Figure S4. HR-TEM images of SnO_2 nanospheres annealed at 500 °C, indicating thicker wall of 25~30 nm containing 6-10 nm interconnected SnO_2 nanoparticles. TEM results are consistent with the average 7.5 nm crystallite size determined from X-Ray diffraction in Figure S5.

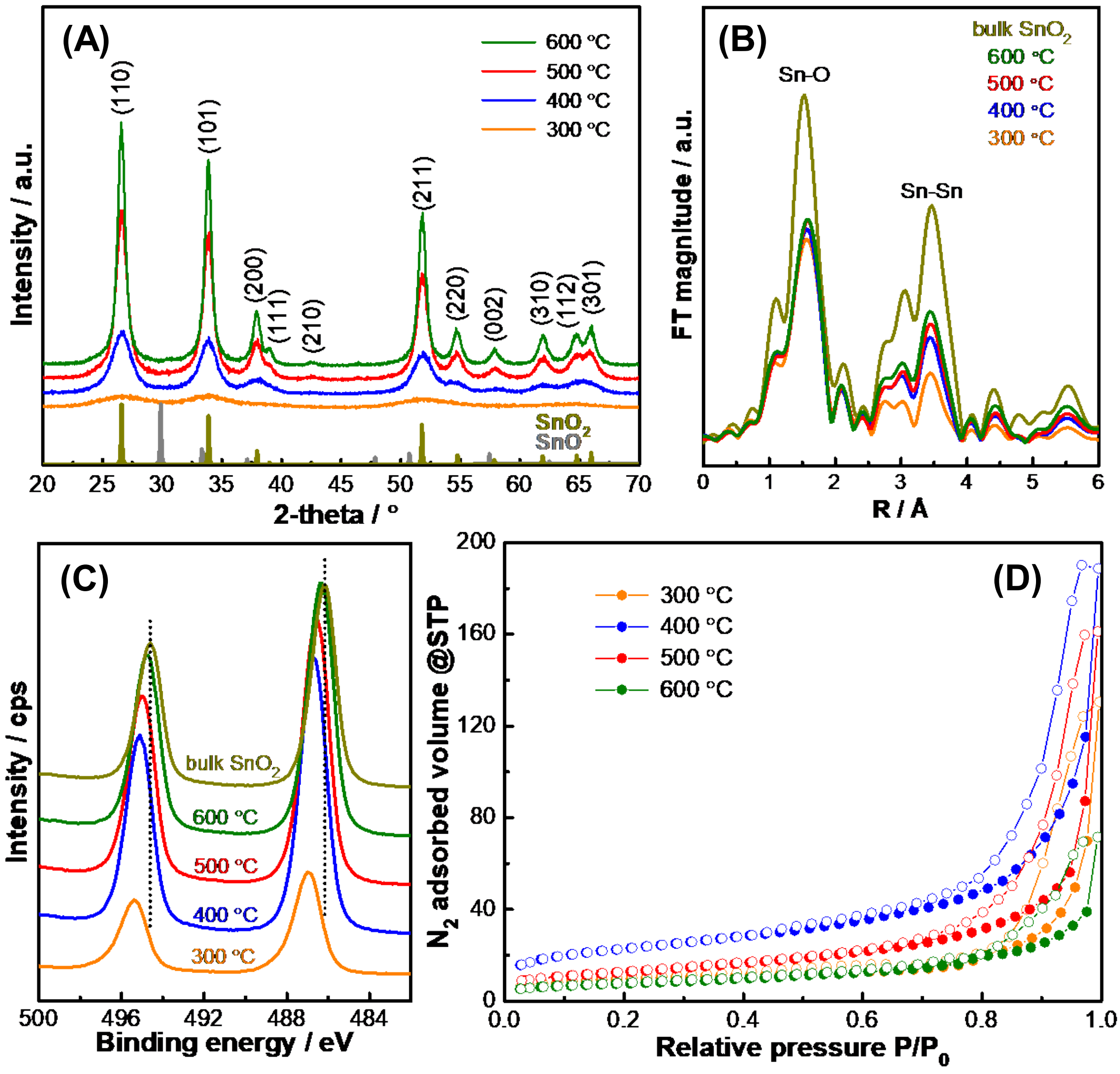

Figure S5. (A) XRD patterns, (B) Fourier-transformed R-space Sn K-edge k^2-weighted EXAFS spectra (not phase corrected), (C) XPS core-level Sn 3d spectra, and (D) N_2 adsorption-desorption isotherms of 3D spherical SnO_2 series. Dark yellow and gray features in (A) represent simulated XRD of SnO_2 and SnO standards for comparison.

The XRD patterns of all 3D SnO_2 nanospheres calcined from 300 to 600 °C in Figure S5A are indexed to pure tetragonal SnO_2 rutile (JCPDS 41-1445) having the space group $P4_2/mnm$. Increasing calcination temperature produced sharper, more intense, peaks that

indicate increased crystallinity and crystallite size up to ~10 nm. In addition, the Sn K-edge EXAFS results in Figure S5B show the presence of first nearest neighbor shell of Sn-O and second Sn-Sn coordination shell for all SnO_2 sphere samples. Higher calcination temperature led to more intense amplitude of these features, further indicating increased crystallinity, particle size, and coordination numbers, with less disorder. The symmetrical Sn $3d_{5/2}$ and Sn $3d_{3/2}$ doublet in Figure S5C corresponds to Sn^{4+} oxidation state in rutile SnO_2. The SnO_2 nanospheres showed up-shifted Sn 3d peaks compared with bulk SnO_2, and lower calcination temperatures (smaller SnO_2 nanocrystals) produced larger binding energy (BE) increases. Similar size-dependent BE shifts have also been observed for other small SnO_2 nanoparticles [36], as well as nanoparticulate Au [37], Pd [38], and PbS [39] systems. Importantly, there was no evidence of Sn^{2+} or any tin-related impurity phases which agreed with XRD results. The BET surface areas obtained from type IV N_2 sorption isotherms (Figure S5D) were 33.6, 79.7, 45.3 and 26.5 m^2 g^{-1} for the nanospheres calcined from 300 to 600 °C.

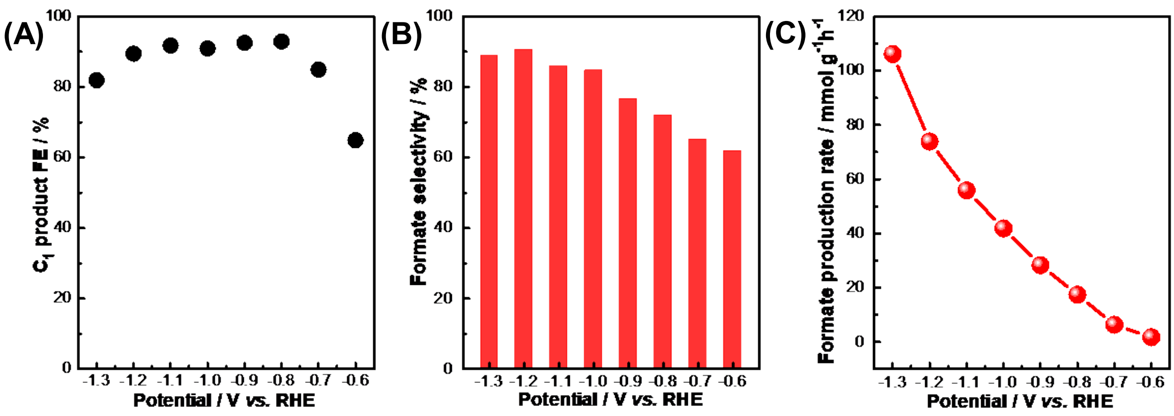

Figure S6. (A) FEs for C_1 products, (B) formate selectivity (referring to total CO_2RR products), and (C) formate production rate for SnO_2 nanospheres calcined at 500 °C.

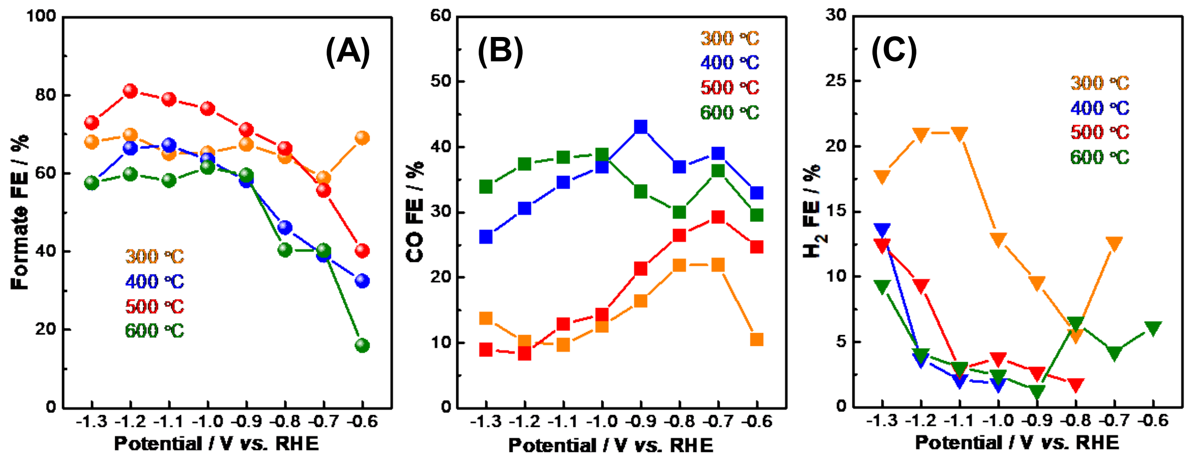

Figure S7. Faradaic efficiencies for (A) formate, (B) CO, and (C) H_2 *vs.* potentials for SnO_2 nanospheres calcined from 300 °C to 600 °C.

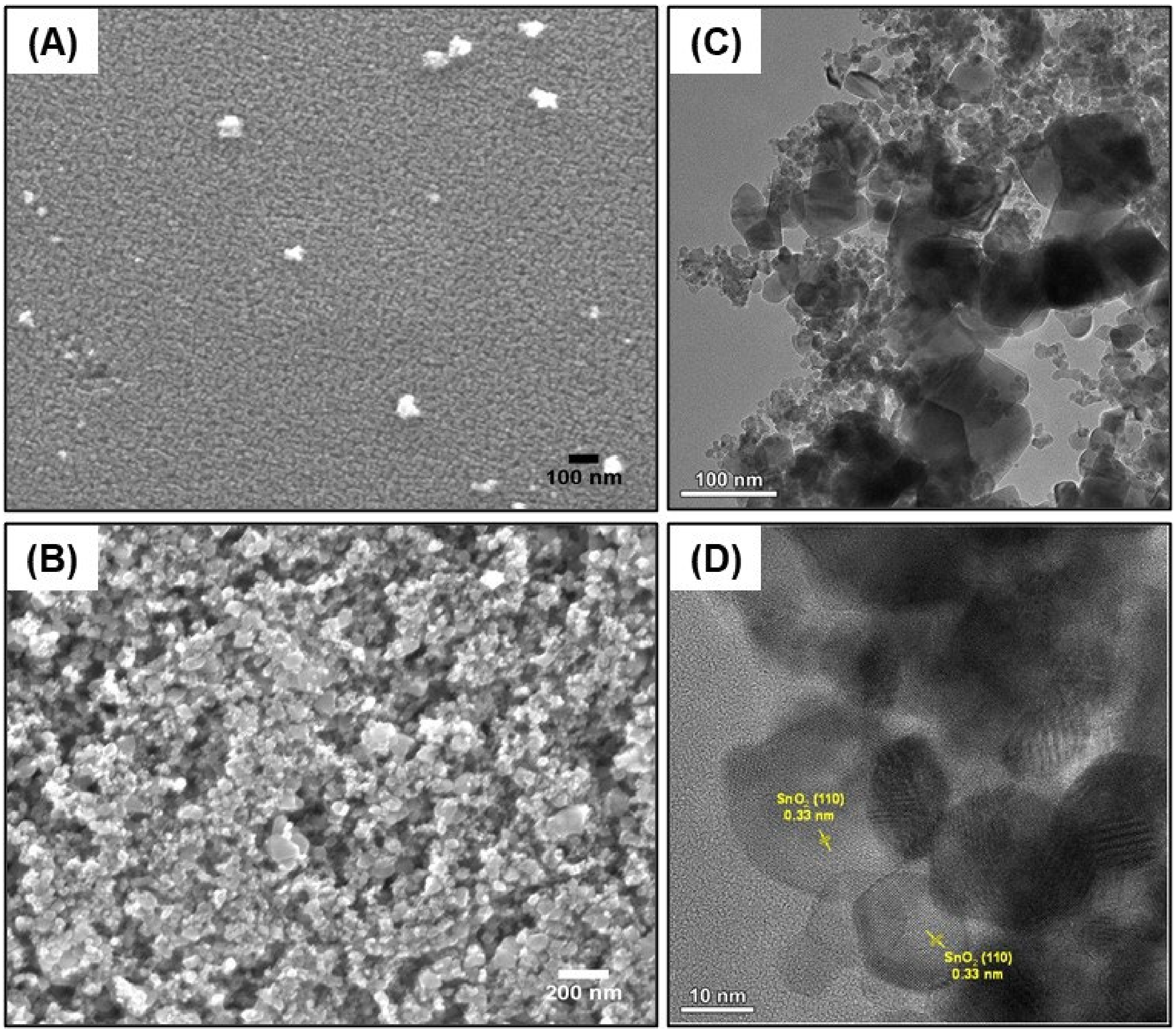

Figure S8. (A, B) FE-SEM images of (A) non-templated SnO_2 nps and (B) com-SnO_2 nps. (C, D) HR-TEM micrographs of com-SnO_2 nps.

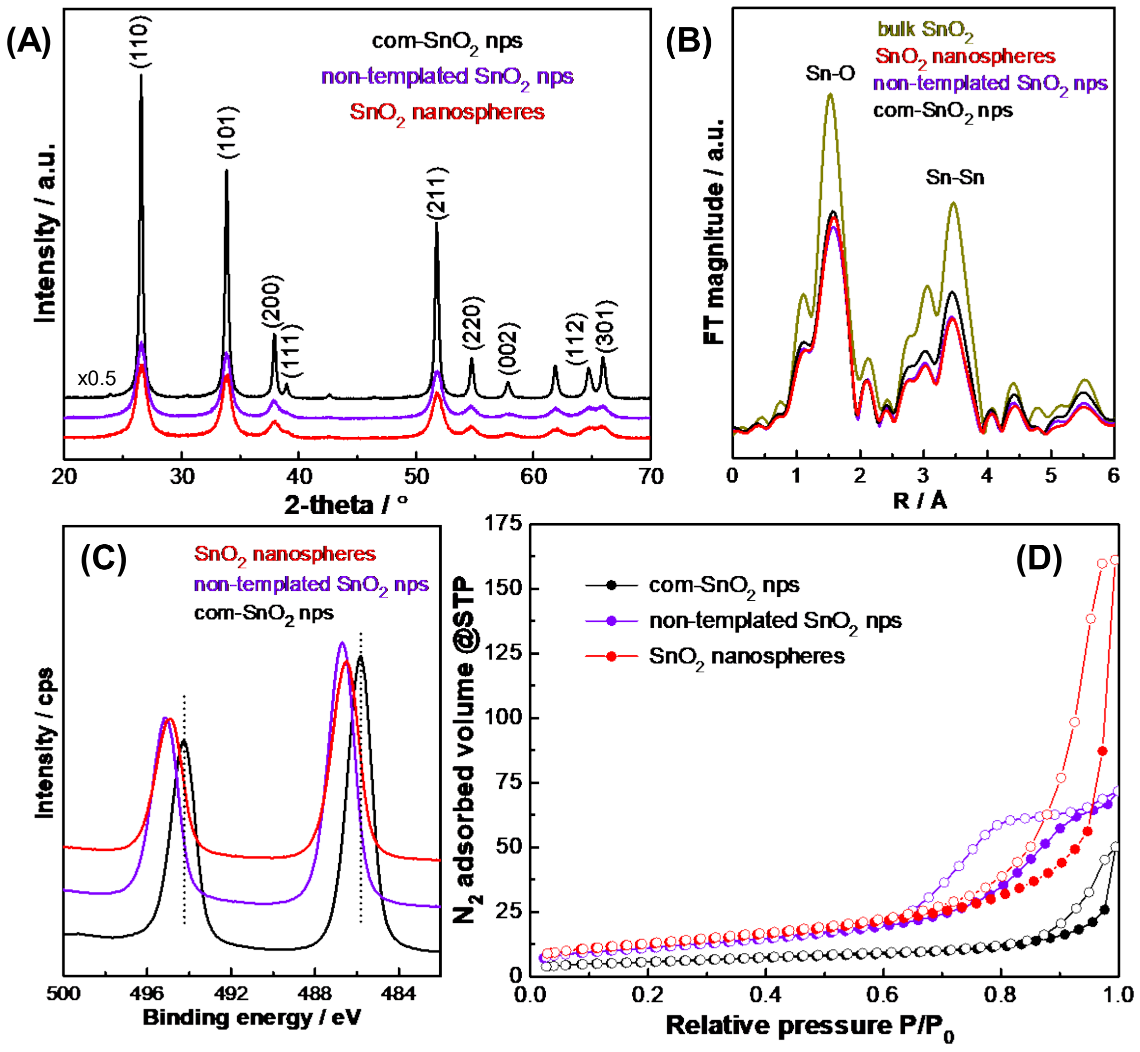

Figure S9. (A) XRD patterns, (B) Sn K-edge EXAFS (not phase corrected), (C) XPS Sn 3d spectra, and (D) N_2 adsorption/desorption isotherms of non-templated SnO_2 nps and com-SnO_2 nps compared with SnO_2 nanospheres calcined at 500 °C.

XRD results in Figure S9A identify the tetragonal rutile SnO_2 crystal structure of all samples. Non-templated SnO_2 nps had almost identical crystallinity, orientation, and crystallite size (~ 7 nm) as hierarchical SnO_2 nanospheres prepared at same temperature (500 °C). However, commercial SnO_2 nanoparticles possessed 4.4 wt% orthorhombic SnO_2 phase (JCPDS 78-1063, space group P*bcn*), a much larger crystal size (*ca.* 28 nm). Similarly, Sn K-edge EXAFS spectra also showed the first nearest neighbor shell of Sn-O and second Sn-Sn

coordination shell of SnO_2 for two nanoparticle samples. The XPS Sn 3d doublets in Figure S9C indicated the presence of Sn^{4+} valence state in both nanoparticle samples. The offset binding energy of commercial sample could be due to much larger particle size. Figure S9D shows type IV nitrogen sorption isotherms of all samples and SnO_2 nanospheres exhibited much larger BET surface area than nanoparticle catalysts (Table S2).

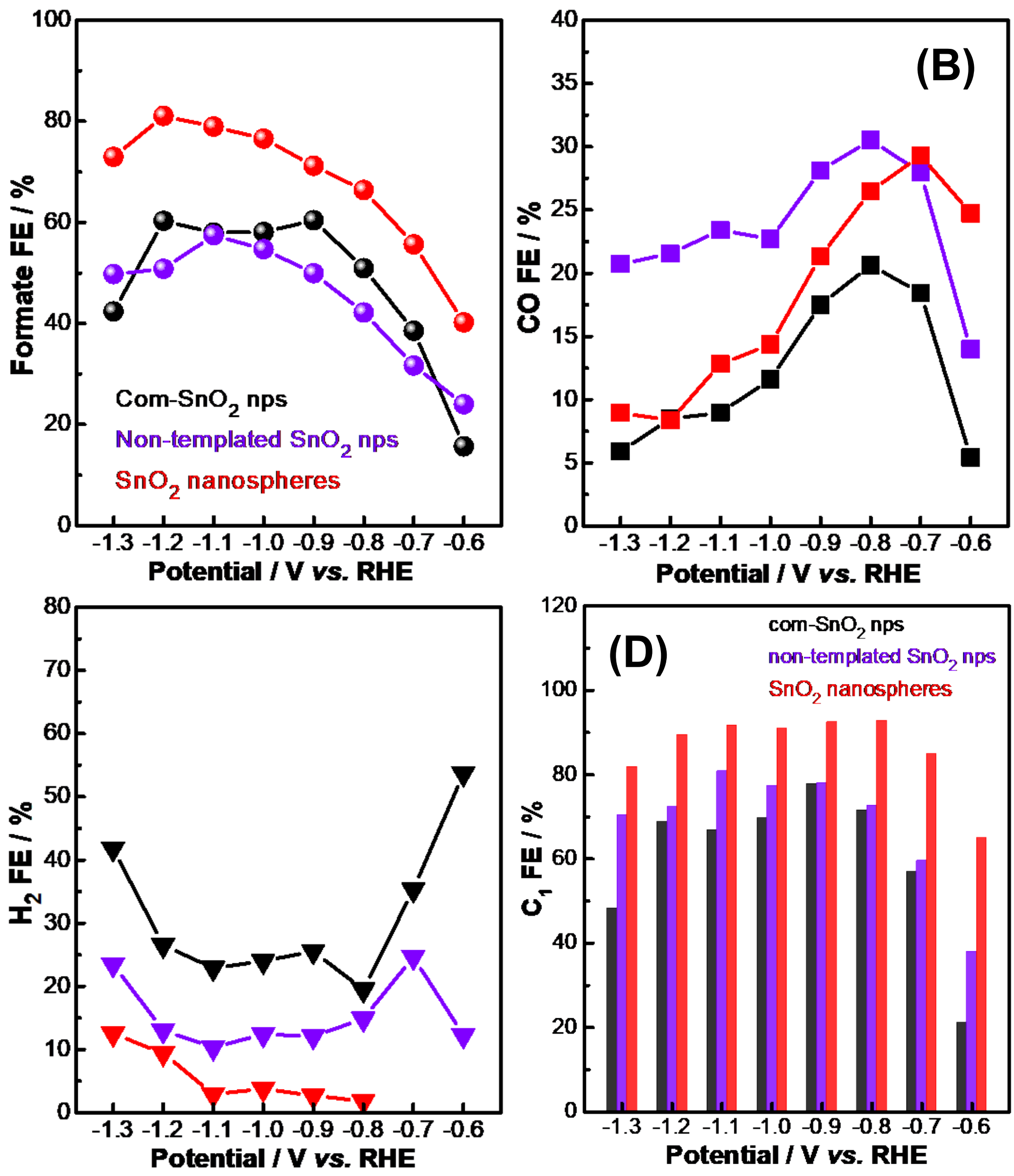

Figure S10. Potential-dependent Faradaic efficiencies for (A) formate, (B) CO, and (C) H_2 of the best performing SnO_2 nanospheres, non-templated SnO_2 nps, and com-SnO_2 nps. (D) Comparison of FEs for C_1 products for three electrocatalysts.

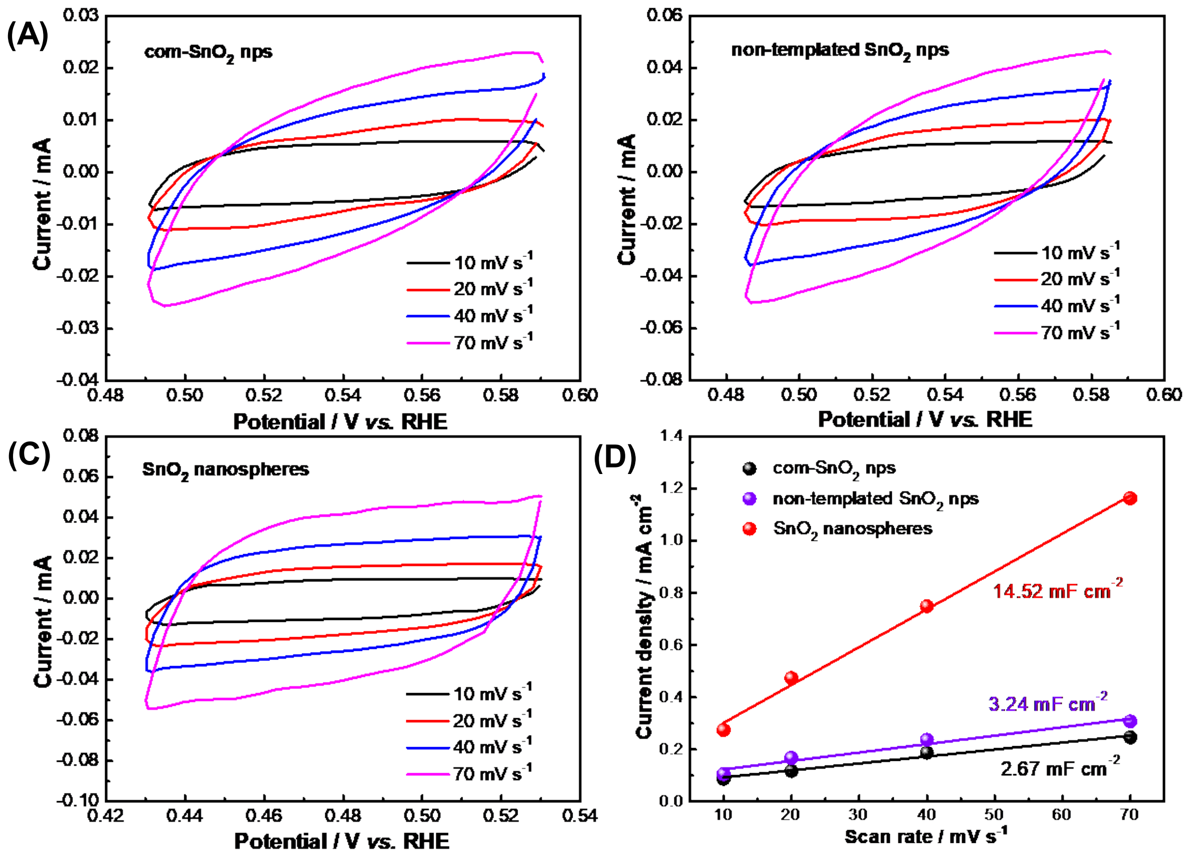

Figure S11. Double-layer capacitance measurement in CO_2-purged 0.1 M $KHCO_3$ electrolyte: (A-C) Cyclic voltammetry profiles measured in the non-Faradaic region with different scan rates, and (D) Scan rate dependence of the current density for com-SnO_2 nps, non-templated SnO_2 nps and SnO_2 nanospheres on Toray carbon paper electrodes. ECSA values are summarized in Table S2.

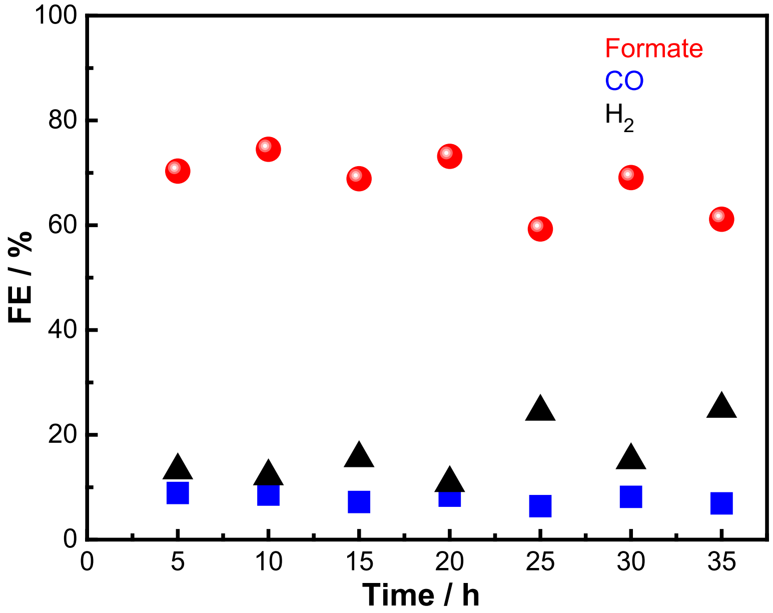

Figure S12. Long-term FEs for formate, CO and H_2 vs. time for SnO_2 nanospheres at -1.2 V *vs.* RHE over multiple 5-hour electrolysis periods.

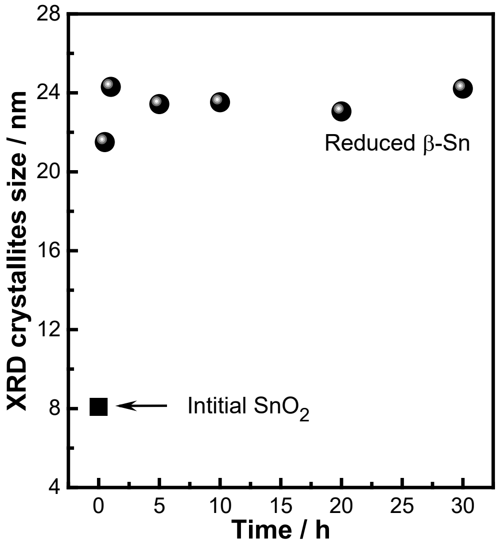

Figure S13. XRD crystallite size of starting SnO_2 and reduced β-Sn as a function of time obtained from time-resolved XRD patterns of SnO_2 nanospheres collected at -1.2 V *vs.* RHE.

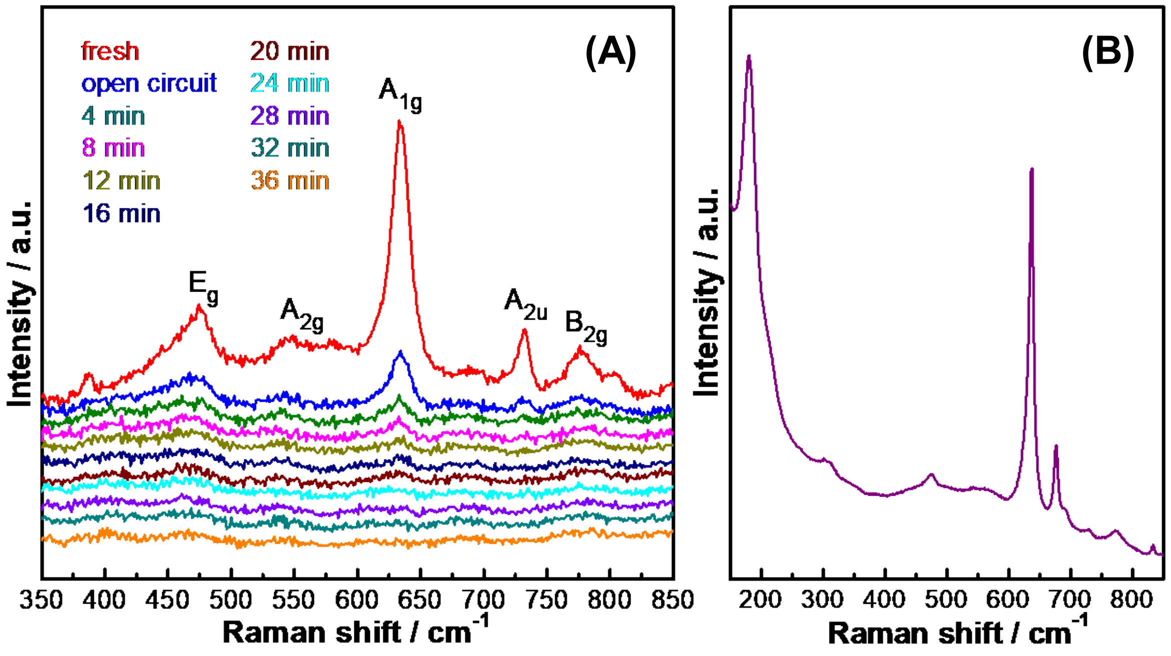

Figure S14. (A) *In situ* time-dependent Raman spectra of SnO_2 nanospheres calcined at 500 °C (on glassy carbon electrode) under CO_2RR at -1.2 V *vs.* RHE. (B) Raman spectrum of the electrode collected under open circuit after CO_2RR at -1.2 V *vs.* RHE.

In situ Raman spectroscopy was conducted to determine the change in oxidation state of SnO_2 nanospheres during application of an electrochemical potential relevant to CO_2RR. Fresh SnO_2 nanospheres catalysts deposited on a glassy carbon electrode (red curve in Figure S14A) show characteristic A_{1g}, B_{2g}, E_g, and A_{2g} Raman modes for rutile SnO_2 (space group D_{4h}). Time-resolved Raman spectra collected at -1.2V *vs.* RHE showed the attenuation of these characteristic bands and then complete disappearance. This result is consistent with the time-dependent XRD shown in Figure 3F (main text) and provides further evidence for the reduction of SnO_2 into metallic Sn during CO_2RR. No other peaks associated with reduced tin oxides and/or surface-bound intermediate species were observed in the wide region of 150-850 cm^{-1}. Our observation is consistent with *in situ operando* Raman results for reduced graphene oxide supported SnO_2 reported by Dutta et al. [40] where oxide fingerprints completely disappeared at very negative potentials, particularly -1.55 V *vs.* Ag/AgCl, as the

catalyst fully reduced to metallic Sn. We additionally found the re-emergence of characteristic SnO_2 Raman bands when the electrode was held at open circuit after electrolysis (Figure S14B), indicating the re-oxidation of metallic Sn into oxide species.

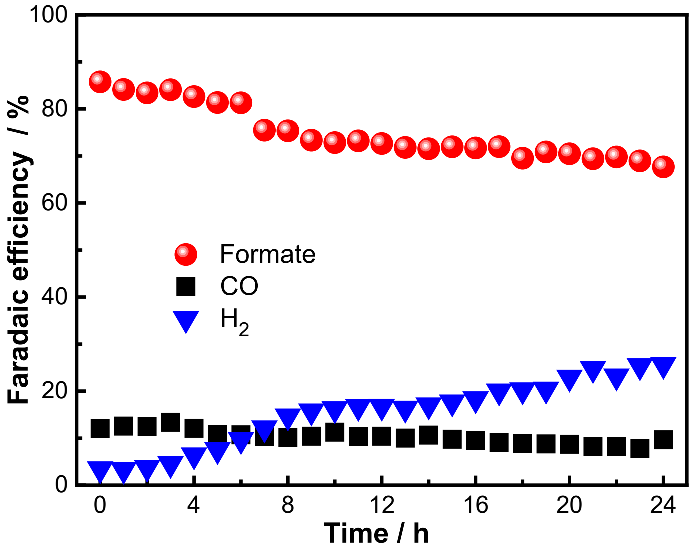

Figure S15. FEs for formate, CO and H_2 during 24-h stability run at 500 mA cm_{geo}^{-2} of SnO_2 nanospheres GDE in 25 cm^2 MEA cell. All testings were performed in aqueous 0.4 M K_2SO_4 catholyte and aqueous 1 M KOH anolyte.

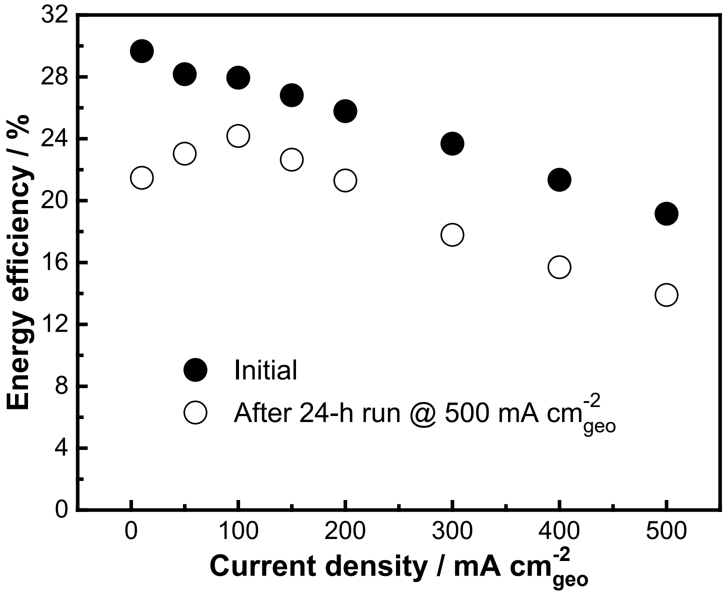

Figure S16. Energy efficiency for formate production as a function of total cell current density.

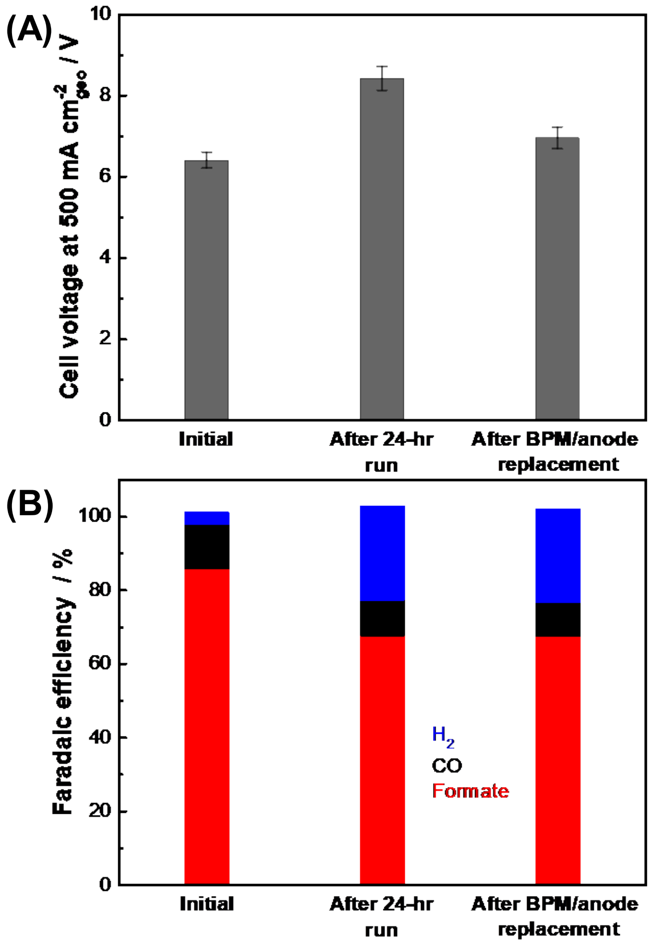

Figure S17. Comparison of (A) cell voltage and (B) FEs for CO, H_2 and formate recorded at 500 mA cm_{geo}^{-2} (left bar), after 24 hours of operation at 500 mA cm_{geo}^{-2} (center bar), and after replacing the BPM and anode (right bar).

The initial polarization data shows the cell required 6.4±0.2V to achieve 500 mA cm_{geo}^{-2}. Subsequent polarization after 24-hours of constant operation at 500 mA cm_{geo}^{-2} required 8.4±0.3V to achieve 500 mA cm_{geo}^{-2}. Replacing the anode and BPM reduced the required cell voltage to 7.0±0.3V at 500 mA cm_{geo}^{-2} in the final polarization curve, which indicates a large fraction of the voltage increase during 24-hour operation was associated with the BPM and anode.

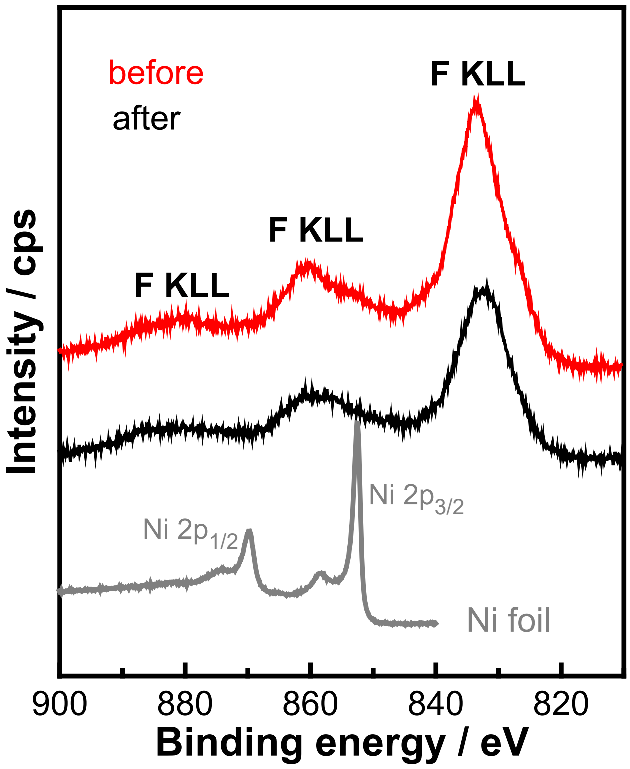

Figure S18. XPS Ni 2p - F KLL Auger spectra of SnO_2 nanospheres GDE before and after CO_2RR in electrolyzer (Ni 2p spectrum of Ni foil standard was included for comparison). The strong Fluorine F KLL features originate from the Nafion binder used in this study, and no distinguishable Ni 2p features were superimposed on the post-reaction F KLL Auger region.

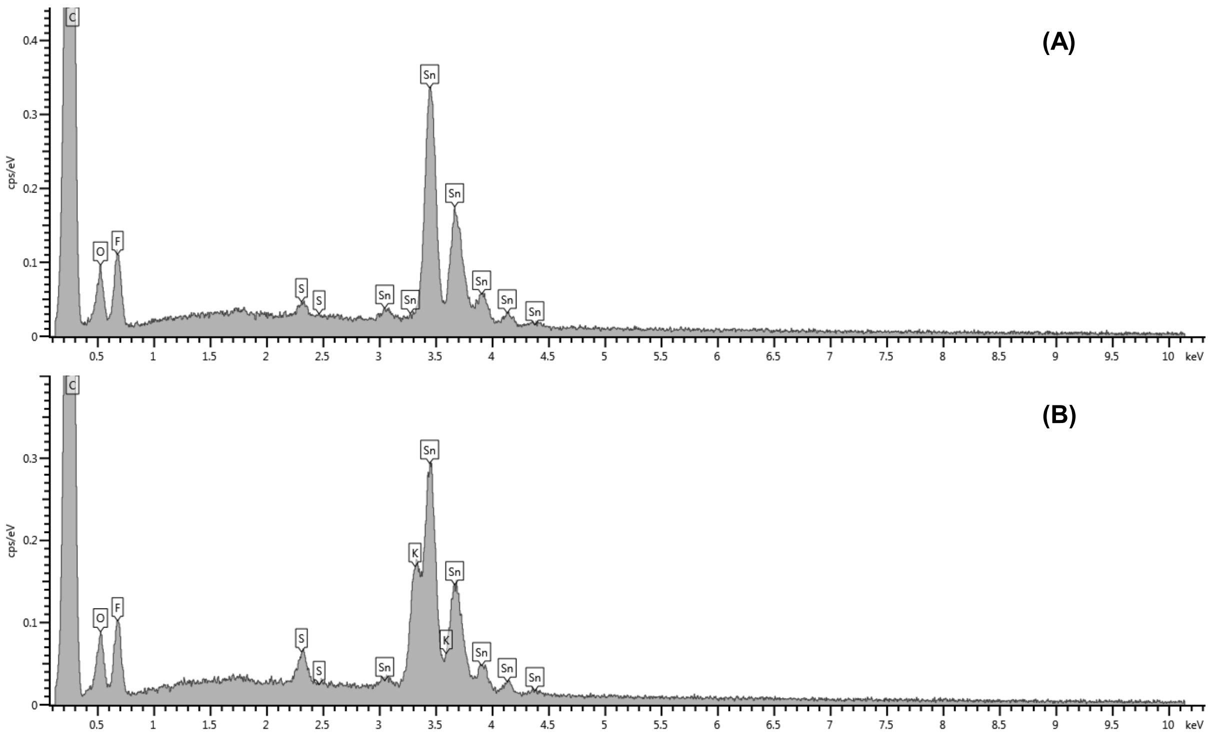

Figure S19. EDX spectra of SnO_2 nanosphere GDE (A) before and (B) after CO_2RR in electrolyzer. K, S and F peaks are from Nafion binder and residual K_2SO_4 catholyte. No signal of Ni contamination was detected.

References

[1] Ravel, B. & Newville, M. *J. Synchrotron Rad.* **12,** 537 (2005).

[2] Liu, S. *et al. Angew. Chem. Int. Ed.* **58,** 8499 (2019).

[3] Morrison, A. R. T. *et al. J. Electrochem. Soc.* **166,** E77-E86 (2019).

[4] Chen, Y. & Kanan, M. W. *J. Am. Chem. Soc.* **134,** 1986 (2012).

[5] Won, D. H. *et al. ChemSusChem* **8,** 3092 (2015).

[6] Lv, W., Zhang, R., Gao, P. & Lei, L. *J. Power Sources* **252,** 276 (2014).

[7] Li, Y. N. *et al. ChemElectroChem* **3,** 1618 (2016).

[8] Du, D. *et al. ChemistrySelect* **1,** 1711 (2016).

[9] Bejtka, K. *et al. ACS Appl. Energy Mater.* **2,** 3081 (2019).

[10] Liu, Y. *et al. Electrochim. Acta* **248,** 123 (2017).

[11] Kumar, B. *et al. Angew. Chem. Int. Ed.* **56,** 3644 (2017).

[12] Daiyan, R. *et al. Adv. Sci.* **6,** 1900678 (2019).

[13] Fan, L., Xia, Z., Xu, M., Lu, Y. & Li, Z. *Adv. Funct. Mater.* **28,** 1706289 (2018).

[14] Liang, C. *et al. J. Mater. Chem. A* **6,** 10313 (2018).

[15] Yadav, V. S. K., Noh, Y., Han, H. & Kim, W. B. *Catal. Today* **303,** 276 (2018).

[16] Gu, J., Heroguel, F., Luterbacher, J. & Hu, X. *Angew. Chem. Int. Ed.* **57,** 2943 (2018).

[17] Yu, J., Liu, H., Song, S., Wang, Y. & Tsiakaras, P. *Appl. Catal. A Gen.* **545,** 159 (2017).

[18] Zhang, S., Kang, P. & Meyer, T. J. *J. Am. Chem. Soc.* **136,** 1734 (2014).

[19] Zhao, C., Wang, J. & Goodenough, J. B. *Electrochem. Commun.* **65,** 9 (2016).

[20] Pavithra, K. & Kumar, S. M. S. *Catal. Sci. Technol.* **10,** 1311 (2020).

[21] Li, F., Chen, L., Knowles, G. P., MacFarlane, D. R. & Zhang, J. *Angew. Chem. Int. Ed.* **56,** 505 (2017).

[22] Zhang, Q. *et al. ChemSusChem* **12,** 1443 (2019).

[23] Lai, Q., Yuan, W. Y., Huang, W. J. & Yuan, G. Q. *Appl. Surf. Sci.* **508,** 145221 (2020).

[24] Zhang, X. X. *et al. Nanoscale* **11,** 18715-18722 (2019).

[25] Liu, H. *et al. GreenChE.* **3,** 138-145 (2022).

[26] Wei, F. *et al. Adv. Funct. Mater.* **30,** 2002092 (2020).

[27] Lei, F. *et al. Nat. Commun.* **7,** 12697 (2016).

[28] Chen, Z. *et al. Appl. Catal. B: Environ.* **261,** 118243 (2020).

[29] Wu, J. *et al. Nano Res.* **14,** 1053-1060 (2021).

[30] Yang, H., Kaczur, J. J., Sajjad, S. D. & Masel, R. I. *J. CO_2 Util.* **20,** 208-217 (2017).

[31] Del Castillo, A. *et al. Appl. Energy* **157**, 165-173 (2015).

[32] Del Castillo, A. *et al. J. CO_2 Util.* **18**, 222-228 (2017).

[33] De Mot, B., Hereijgers, J., Daems, N. & Breugelmans, T. *Chem. Eng. J.* **428**, 131170 (2022).

[34] Merino-Garcia, I. *et al. Appl. Catal. B* **297**, 120447 (2021).

[35] Chen, Y. *et al. ACS Energy Lett.* **5,** 1825-1833 (2020).

[36] Ahn, H.-J., Choi, H.-C., Park, K.-W., Kim, S.-B. & Sung, Y.-E. *J. Phys. Chem. B* **108,** 9815 (2004).

[37] Peters, S., Peredkov, S., Neeb, M., Eberhardt, W. & Al-Hada, M. *Surf. Sci.* **608,** 129 (2013).

[38] Wang, H. *et al. Sci. Adv.* **5,** eaat6413 (2019).

[39] Choi, H., Ko, J.-H., Kim, Y.-H. & Jeong, S. *J. Am. Chem. Soc.* **135,** 5278 (2013).

[40] Dutta, A. *et al. Nano Energy* **53,** 828 (2018).
